# There Is No Place Like Home: The Impact of Public Home‐Based Care on the Mental Health and Well‐Being of Older People

**DOI:** 10.1002/hec.4948

**Published:** 2025-02-20

**Authors:** Ludovico Carrino, Erica Reinhard, Mauricio Avendano

**Affiliations:** ^1^ Department of Economics, Business, Mathematics and Statistics “Bruno de Finetti” University of Trieste Trieste Italy; ^2^ Department of Global Health & Social Medicine King's College London London UK; ^3^ Department of Epidemiology and Health Systems Center for Primary Care and Public Health (Unisanté) University of Lausanne Lausanne Switzerland; ^4^ Department of Social and Behavioral Sciences Harvard T. H. Chan School of Public Health Boston Massachusetts USA

**Keywords:** depression, formal care, home care, instrumental variable, long‐term care, mental‐well‐being, psychological well‐being, public policy

## Abstract

Despite a significant policy shift from institutional to home‐based care for older adults, evidence on the effectiveness of policies incentivizing home care is limited. This study provides novel evidence on the causal effect of public home‐based care on the mental health and well‐being of older people. To address endogenous selection, we implement a novel instrumental variable approach that exploits eligibility rules for long‐term care as defined in national legislations. We link longitudinal data from the Survey of Health, Aging & Retirement in Europe (SHARE, 2004–2017) to national LTC eligibility rules in France, Germany, Spain and Belgium (disaggregated for Wallonia and Flanders regions) and examine how exogenous variation in the use of long‐term care caused by varying eligibility rules impacts depressive symptoms (EURO‐D scale), quality of life (CASP scale) and loneliness (R‐UCLA scale). We find that receiving formal home‐based care significantly reduces depressive symptom scores by 2.6 points (large effect size measured by Cohen's d) and the risk of depression by 13 percentage points. The use of home‐based formal care also increases quality of life as measured by the CASP scale, particularly by increasing feelings of control over life. We show that one potential mechanism involves the impact of home‐based care on loneliness: we estimate that receiving formal home‐based care reduces the risk of loneliness by 6.7 percentage points. Our results provide evidence that an increase in home‐based care coverage is justified in terms of improved mental health and well‐being outcomes for older people.

## 1 Introduction

Rapid demographic aging has increased concerns about the sustainability of Long‐Term Care (LTC) systems. OECD countries have witnessed a rapid increase in the demand for LTC services by vulnerable older people, which is predicted to increase public expenditure for LTC (compared to year 2019) by 41% in the next 20 years, and by 64% by the year 2070 (European Commission 2021). An increasingly common policy response to the rising demand for LTC is “aging‐in‐place,” an approach that promotes enabling older adults to remain in their community with some degree of independence, supported by home‐based services, rather than moving to residential care (Davey et al. 2004). In line with this approach, most European countries have increasingly prioritized the provision of formal home‐based care (WHO 2015), with a focus on subsidized services for vulnerable older people. Critical to this policy shift is the assumption that home‐based care delays functional decline and improves well‐being, thus reducing costs and improving quality of life (Hashiguchi and Llena‐Nozal 2020). However, empirical evidence on whether home‐based LTC improves outcomes remains limited. Establishing a causal relationship is particularly challenging due to potential endogeneity, driven by reverse causality and omitted variables bias.

This study provides novel evidence on the causal effect of publicly funded home‐based care on mental health and quality of life, focusing on four European countries with well‐established LTC systems. We focus on depression, a highly prevalent mental health problem in older age estimated to affect 12% of adults aged 65+ in Europe (Purebl et al. 2015). Depressive symptoms are common among older adults with activity restrictions and functional limitations (Penninx et al. 1998; Williamson and Shaffer 2002), and have been linked to declines in cognitive and physical functioning (Ormel et al. 2002). Mental health disorders are a major cause of disability, negatively affecting economic outcomes including employment and social participation (James et al. 2018; Purebl et al. 2015), with economic costs estimated at 4% of GDP in OECD countries (OECD/EU 2018).

The impact of home LTC on mental health is ambiguous. On the one hand, formal home‐based care may improve mental health through greater flexibility in leisure time‐allocation, consumption and living arrangement decisions. Home care may also address unmet need, help maintain independence, autonomy and social participation, and reduce loneliness (WHO 2015), potentially also reducing the burden of informal carers. On the other hand, home care may reduce self‐sufficiency, increase dependency at home, reduce independence, and increase emotional distress for older persons and their family (Kwak, Ingersoll‐Dayton, and Burgard 2014; Silverstein, Chen, and Heller 1996). Empirical evidence, therefore, is essential to establish whether policies targeted toward home care can improve welfare while containing costs.

In this paper, we use an instrumental variable approach that exploits the nonlinearity and heterogeneity of eligibility rules for public LTC in four European countries. In order to receive public LTC support, older people need to fulfill eligibility criteria, which are a nonlinear aggregation of functional and cognitive limitations and which vary substantially across countries (Brugiavini et al. 2017; Gori and Fernandez 2015). This implies that two otherwise similar individuals may differ in their eligibility for home‐based care due to slight variations in their functional limitations, or differences in eligibility criteria.

We link longitudinal data from the Survey of Health, Aging & Retirement in Europe (SHARE, 2004–2017) to novel data on LTC legislation in France, Germany and Spain and Belgium (disaggregated for Wallonia and Flanders regions) (Brugiavini et al. 2017). We use an instrumental variable approach that exploits the nonlinearity and heterogeneity of LTC rules. Our identification strategy uses an individual‐level variable identifying respondents' eligibility for public home care as instrument for formal home care use in a 2SLS model. While the likelihood of receiving care rises with worsening health and functional limitations, country‐specific idiosyncrasies in LTC eligibility rules create potentially exogenous variation in the probability of receiving home‐based long‐term care. Conditional on health and regional fixed effects, our instrument captures exogenous variation in the probability of receiving care arising from local eligibility rules. Our instrument, which is grounded in legislation‐driven eligibility criteria, strongly predicts the probability of formal care use. We measure psychological well‐being using the validated Euro‐D depression scale, the CASP scale for quality of life, and the R‐UCLA scale for loneliness.

Our study makes two important contributions to the existing literature. First, we show that a policy approach that favors and finances formal home‐based LTC can substantially improve the mental health and life satisfaction of older people: in our preferred specification, receiving formal home‐based care reduces depressive symptom scores by 2.6 points (a large effect size as measured with the Cohen *d*) and the risk of depression by 13 percentage points. Receiving formal home care increases the probability of reporting higher than average Quality of Life scores by 15 percentage points, through an increase in feelings of control over life; and it reduces the risk of loneliness by 6.7 percentage points. Second, we show how differences in legislation can be used to estimate the causal effect of care, a novel approach that can be extended to examine the welfare impact of health and LTC (Pestieau and Lefebvre 2018). Our approach improves upon previous studies which either lacked a source of exogenous variation in the use of LTC or relied on instruments with less defensible exclusion restrictions.

Our results have important policy implications. First, they suggest that the net effect of home‐based LTC on mental health and quality of life is positive and large. Second, they support calls for more inclusive eligibility criteria for home‐based LTC and suggest that budget cuts to LTC services should factor in possible welfare losses for older people. Third, they suggest that a policy‐driven increase in home‐based care coverage can provide a valuable tool to reduce common mental health disorders, reduce loneliness and improve quality of life in older age.

Our paper proceeds as follows. Section 2 discusses the causal mechanisms between LTC and mental health. Section 3 outlines our empirical framework and contribution to the literature. Section 4 explains our instrumental variable approach and describes our data. Results are outlined in Section 5, while Section 6 concludes with a discussion.

## 2 Conceptual Framework and Institutional Context

What are the consequences of receiving formal care on well‐being? Existing theoretical frameworks offer mixed and sometimes conflicting hypotheses.

Models of long‐term care demand, supply and financing assume a positive causal link between care use and utility (see, e.g., Nuscheler and Roeder 2013; Stabile, Laporte, and Coyte 2006; Van Houtven and Norton 2004; J. Forder et al. 2018 and the review by Barnay and Juin (2016)). In these models, utility is conceived as an increasing function of health, which is in turn assumed to be an increasing function of (formal or informal) care services and human capital. The marginal productivity of care is assumed to be positive, although the returns can be increasing, decreasing or constant in the amount of care, depending on type of inputs, care setting or level of needs. For example, Kuhn and Nuscheler (2011) and J. Forder et al. (2018) assume that a unit of care is more productive at worse levels of health.

Sen's *capability model* of well‐being (Nussbaum and Sen 1993) characterizes a person's life as a combination of valuable “doings and beings” (*functionings*), where well‐being is determined by a person's freedom to choose from a set of such combinations (*capabilities*) (Stiglitz, Sen, and Fitoussi 2010). Accordingly, J. E. Forder and Caiels (2011) theorize that receiving formal care improves psychological well‐being by increasing capabilities. Home‐based care may *directly* reduce or prevent functional decline and activity restriction, for example, by helping them dress and go out, which will reduce the risk of decline in mental well‐being and improve life satisfaction (WHO 2015; Williamson and Shaffer 2002). *Indirectly*, it may increase the capability to achieve other *functionings*, for example, leisure activities, hobbies and social contacts, which will increase enjoyment, control, and self‐respect (Grewal et al. 2006), leading to higher psychological well‐being and life satisfaction.

Recent clinical literature also highlights that activity restriction (the inability to perform activities related to personal independence) caused by physical decline acts as a stressor, which can lead to depressive symptoms and reduced emotional well‐being, by worsening one's sense of self and a loss of control over life (Williamson and Christie 2009; Williamson and Shaffer 2002). Home‐based care may improve psychological well‐being through greater feeling of control and autonomy, for example, from increasing flexibility in leisure time‐allocation, consumption and living arrangement decisions (Grewal et al. 2006).

A potential mechanism for the impact of home‐based care on mental health relates to its social‐connection enhancement role (Berkman et al. 2000; Thomas, Akobundu, and Dosa 2016; Wolff and Agree 2004). As people age, a loss of social connections reduces mental health and increases feelings of loneliness, especially for people with activity restrictions (S. Cohen 2004; Deci and Ryan 2000; Weiss 1973). Sociological theories predict that the interpersonal transfers occurring during home visits (including related services such as meals‐on‐wheels) restores or improves social interactions. Through this mechanism, home‐based care may reduce loneliness, mitigate the negative effect of physical health deterioration, and improve mental well‐being (Broese van Groenou 2020; Donovan et al. 2017; Thomas, Akobundu, and Dosa 2016; Wolff and Agree 2004).

### 2.1 Alternative Hypothesis: Care Use Worsens Psychological Well‐Being

On the other hand, home‐based care may have negative consequences for well‐being. First, it may reduce older people's incentives for self‐sufficiency, leading to loss of skills for independent living, thus increasing vulnerability and reducing autonomy (Silverstein, Chen, and Heller 1996). *Identity Theory* and *Self‐Determination Theory* (e.g., Stets and Turner 2014; Deci and Ryan 2000) predicts that self‐reliance and autonomy are highly valued by individuals, and may be negatively affected by the receipt of care at home (Kwak, Ingersoll‐Dayton, and Burgard 2014; Roe et al. 2001). Formal care receipt may also foster negative self‐perceptions of aging, which may increase depression and reduce well‐being (Kwak, Ingersoll‐Dayton, and Burgard 2014; Silverstein, Chen, and Heller 1996).

Second, home‐based care may influence the well‐being of adult children and other informal carers, which can in turn affect older people's well‐being. If home‐based care serves as a substitute for informal care, an increase in the supply of formal home‐based care is likely to reduce the amount of care provided informally. This will reduce the burden of long‐term care and improve the well‐being of family and friends, with positive spillover effects on the well‐being of older people. However, there are at least two reasons why these effects may not materialize. First, if older people's preferences favor care by family members rather than formal carers, a substitution of informal support by home‐based care may increase feelings of social isolation and loneliness, leading to higher risk of depressive symptoms and poorer well‐being. Second, recent literature suggests that, except for highly specialized care and cases of severe loss of autonomy (Bolin, Lindgren, and Lundborg 2008; Bonsang 2009), formal care is complementary—rather than substitute—to informal care. Theoretical models predict that an increase in the availability of formal care through public provision would reduce informal care provision only when care needs were already fully met by all unpaid (public and family) sources of care (Stabile, Laporte, and Coyte 2006). Conversely, in situations where care needs are not fully met, higher public LTC provision would generate an income effect, freeing resources that can be used to compensate informal caregivers.

Overall, studies in Europe indicate that the utilization of formal and informal care is increasingly complementary. Consequently, an increase in home‐based care is expected to result in a marginal increase in informal care provided by friends and families, and vice versa. In addition, as shown by Carrino, Orso, and Pasini (2018), when overall care demand is unmet by either formal or informal sources, increased formal care use can lead to marginally higher informal care use (Balia and Brau 2013; Bonsang 2009; Carrino, Orso, and Pasini 2018). Based on this evidence, we hypothesise that an increase in home‐based formal care would reduce unmet need and improve the well‐being and mental health of older people.

### 2.2 Context: LTC Systems in Four European Countries

Our study focuses on Belgium, France, Germany and Spain, as these countries have a system of LTC based on an assessment of needs to provide public LTC support for older people, using algorithm‐based eligibility rules. Specific features of their systems vary across countries: for example, the French and Belgian systems are mostly in‐kind, while the German and Spanish systems give a choice between in‐kind and in‐cash benefits, which can be mixed in Germany yet not in Spain (Barber, Ong, and Han 2021). The broader policy context for LTC varies greatly across these countries. For example, the OECD Health Statistics 2023 report that total public spending on LTC as a percentage of GDP (in 2021) ranged from to 3% in Belgium, to 2.5% in France and Germany, and to 1% in Spain. Higher spending does not necessarily mean a higher protection for the average user, for example, because the costs of receiving LTC are not homogeneous across countries. Hashiguchi and Llena‐Nozal (2020) computed the costs of receiving home‐care care for an individual with severe needs (requiring 40 h of care per week), in absence of public support, compared to the average disposable income of the population aged 65+. They estimate that, in France, LTC costs would amount to 300% of the average income, while for Belgium (Flanders), Germany and Spain the share of the costs would be around 200%. Moreover, they have computed the share of individual costs that would be subsidized by public LTC programmes for eligible individuals with severe needs, earning a median income and holding no net wealth. Unsurprisingly, different public LTC systems are expected to cover very different shares of LTC costs. In Belgium (Flanders) and Germany, they estimate that around more than 80% of home‐care costs for someone with severe needs would be covered by the public social protection system; such percentage drops to around 55% for France and Spain.

## 3 Empirical Framework

Recent country‐specific studies have linked the availability of home care to improvements in welfare, through reductions in hospitalizations and increased survival (Costa‐Font, Jimenez‐Martin, and Vilaplana 2018; Hernández‐Pizarro 2016; Orsini 2019; Rapp, Chauvin, and Sirven 2015). However, there is little evidence of impacts on mental health, quality of life and well‐being outcomes. Some studies have explored this question by comparing the well‐being outcomes of care users with those of non‐users, such as Kwak, Ingersoll‐Dayton, and Burgard (2014), Pepin et al. (2017), Andersson and Monin (2017) and Broese van Groenou (2020), and most find a negative association between care use and well‐being. We summarize these studies with the following equation:

$${\text{WB}}_{i}=\alpha {\;+\;\beta \text{FC}}_{i}+\gamma {\text{IC}}_{i}+X\delta +\varepsilon_{I}$$

where WB represents a measure of psychological well‐being, for example, depression score; FC and IC measure, respectively, formal and informal care utilization, and the vector X includes other (present or lagged) individual socio‐demographic and health characteristics. A key challenge is that the receipt of home‐based care is endogenous to both physical and mental health. Therefore, the estimated coefficient *β* (and *γ*) cannot be interpreted as a causal effect, as it is biased by the endogeneity of care decisions (Barnay and Juin 2016). Studies show that having a lower psychological well‐being increases the probability of receiving formal (and informal) care (Portrait, Lindeboom, and Deeg 2000; Stabile, Laporte, and Coyte 2006). In addition, the amount of formal and informal care received is likely to reflect the outcome of a simultaneous decision process involving the dependent person and her network (Stabile, Laporte, and Coyte 2006; Van Houtven and Norton 2004). Likewise, unobserved factors might affect both psychological well‐being and care utilization decisions, such as family health history and individual preferences, which may affect the choice of care and the experience of coping with the stress of limitations in daily activities (J. E. Forder and Caiels 2011; Hu and Wang 2019).

Few studies have attempted to estimate the causal relationship between care use and mental well‐being addressing the endogenous nature of care decisions, with inconsistent findings. Barnay and Juin (2016) use cross sectional data on French adults with functional limitations. They find that informal care—instrumented using characteristics of adult children—reduces the probability of reporting depression in the last 2 months, while formal home‐based care—instrumented using geographical disparities in access to public long‐term care coverage ‐improves overall mental health as measured by Mental‐Health Inventory (MHI‐5), but it does not affect depression. J. Forder et al. (2018) employ data from a sample of public home care users in England with physical disability or sensory/mental impairment and use aggregate‐level data on service use at the local authority level to instrument formal care use. They find that community‐based formal LTC significantly improves care‐related quality of life, with diminishing marginal effects and heterogeneity depending on baseline impairment level (the most impaired individuals benefit the most). Stabile, Laporte, and Coyte (2006) use data from Canada to test whether an increase in the use of publicly subsidized formal care improves self‐reported health. They employ three macro‐level variables as instruments for the generosity of the public home care program: the share of the population aged 65 and older in each province over time; the level of provincial spending on education in each province over time; and the provincial tax rate as a share of federal taxes in each province over time. Their results show no significant impact of care‐use on health.

A key limitation of previous studies is that they all use macro‐level instrumental variables, whereby all individuals from the same area are assigned the same treatment. As LTC policies affect only specific population subgroups, instruments have limited informative power (Bound, Jaeger, and Baker 1995). Moreover, macro‐level instruments such as service or expenditure data raise endogeneity concerns. For example, it is possible that a lower availability of informal caregivers affects the availability of publicly‐funded home care (Golberstein et al. 2009); or that changes in expenditure in LTC are correlated with changes in expenditure in other public services. To address these concerns, some studies focus on specific population subgroups, for example, they restrict the sample to people already using care or already facing functional limitations, potentially introducing selection bias and compromising external validity.

In this paper, we follow a novel approach that exploits detailed information on eligibility for public programmes of home care in four European countries. Using an international micro dataset, we construct a binary eligibility instrument at the individual level that identifies those meeting the minimum requirements for formal LTC in their country or region of residence. We exploit the fact that access to formal home care programmes is not discretionary, but it is characterized by mandatory gateways such as assessment of need and eligibility rules (Brugiavini et al. 2017; Eleftheriades and Wittenberg 2013). Our approach relies on the fact that eligibility criteria are highly heterogeneous across countries and lead to important variation in home‐based care use (Bakx et al. 2014; Carrino, Orso, and Pasini 2018; Costa‐Font, Jimenez‐Martin, and Vilaplana 2018; Da Roit and Le Bihan 2010; Gori and Fernandez 2015; Hashiguchi and Llena‐Nozal 2020; Muir 2017).

## 4 Methods

### 4.1 Econometric Model

In order to estimate the effect of formal‐care use on depression, we start from the following model, for individual *i* living in area *r*:

(1) $${\text{MH}}_{i,r}=\gamma_{0}+\gamma_{1}{\text{FC}}_{i,r}+\gamma_{2}{\text{IC}}_{i,r}+\gamma_{3}^{\prime }{\text{HS}}_{i,r}+\gamma_{4}^{\prime }X_{i,r}+\gamma_{5}{R_{r}+\varepsilon }_{i,r}$$

where MH is individual depression score, and FC and IC are binary indicators for formal‐ and informal‐care use respectively. Based on prior literature (Barnay and Juin 2016; Bolin, Lindgren, and Lundborg 2008; Bonsang 2009; Van Houtven and Norton 2004), we include a vector of health controls (HS), including a binary indicator for poor self‐rated health; number of limitations in Activities of Daily Living (ADL), and in Instrumental ADL (iADL); binary indicators for having any mobility limitation; and having low cognitive function (see Section 4.3.4 for a description of how these variable are coded).^1^ We also include a vector of sociodemographic controls (*X*), including living in a couple, living in a urban or rural setting, highest educational attainment, household income quintile, a second order polynomial for age.

We include fixed‐effects for interview‐year, and for region of residence (*R*) (NUTS1 level) to account for time‐invariant regional characteristics.

### 4.2 Instrumental Variable Approach

We implement a two‐stage least squares instrumental variable (2SLS IV) approach where the outcome variable is the continuous depression score, and the main regressors of interests are binary variables for the receipt of home‐based formal and informal care. We implement an IV probit model in analyses that employ a binary dependent variable. In order to control for potential correlation with unobservables among individuals living in the same region, we cluster standard errors at the NUTS1 level (57 clusters).

In order to instrument home‐based formal care use, we construct an individual‐specific, binary, eligibility index, which identifies individuals whose health profile fulfills the minimum requirements of any LTC program implemented in their region or country of residence. Our identification relies on the fact that each national or regional LTC legislation in Europe has unique and different assessment and eligibility criteria for granting access to LTC formal care (Brugiavini et al. 2017). We therefore exploit the interaction between individual health characteristics and country‐specific legislation for causal identification. While an individual's probability of using formal care increases with the number of functional (and cognitive) limitations, such probability is significantly higher if, *ceteris paribus*, the individual is eligible for LTC support (Bakx et al. 2014; Carrino, Orso, and Pasini 2018). Hence, controlling for individual health conditions and incorporating country and region fixed‐effects, the eligibility instrument leads to exogenous variation in the probability of receiving care.

#### 4.2.1 Properties of the Instrument for Formal Care

We estimate our model on four European countries (Belgium, France, Germany and Spain), because their legislation sets out clear‐cut (algorithm‐based) eligibility rules, their programmes are targeted (monitored) to home‐based care (Brugiavini et al. 2017), and eligibility is *carer*‐*blind*, that is, the need‐assessment excludes availability of informal care (Eleftheriades and Wittenberg 2013). Data on eligibility was collected through revision of legislations and expert interviews in each country, as detailed in Brugiavini et al. (2017). Details of the LTC programmes considered in this paper is available in Supporting Information S1: Appendix 2, together with information on the construction of our instrument (see also Brugiavini et al. 2017).

The eligibility instrument should be informative and exogenous. Informativeness lies on both the non‐linearity of the algorithm and weights embedded in each LTC programme's eligibility rules, and in their variation across‐country and time. The individual eligibility status varies both because of different health conditions across subjects and because of different assessments of the same health conditions across programmes. The eligibility rules are thus non‐linear combinations of applicants' health characteristics, including limitations in physical function (e.g., ADL and iADL) and cognitive performance,^2^ such that only specific combinations of an applicant's health characteristics trigger eligibility for LTC. Moreover, the eligibility procedures differ largely across countries. For example, in order to meet the minimum eligibility threshold for the Belgian INAMI programme, individuals need to be (i) limited in bathing and dressing, and (ii) being disoriented in time and space, or (iii) being further limited in moving around the house or in using the WC. A similar framework is adopted in the national programme in France. By contrast, in Germany, Spain and in the Belgian APA programmes, eligibility is determined by virtue of comparing an individual “need‐of‐care” score against a specific “minimum threshold.” For example, in Germany (before the 2017 reform), eligibility required a minimum daily‐need of 90 min‐of‐help, with at least 45 min attributable to ADL, while cognitive limitations were not part of the assessment until 2012.

In terms of age requirement, there is no minimum age to claim benefits for LTC programmes in Germany or Spain, while there is a minimum age in France (60 years) and the Belgian APA programme (65 years). The public support for in‐kind LTC in Belgium had no minimum age eligibility before 2014, where the minimum age was set to 60 (in Wallonia). For further details, see Brugiavini et al. (2017) and the country profiles made available by the G2AGING project at https://g2aging.org/ltc/long‐term‐care.

The idiosyncratic nature of LTC legislations implies that two individuals with very similar clinical profiles may differ in their eligibility for home‐based care, due the specific combination of their impairments. Likewise, their eligibility might differ by virtue of different legislations if they live in different areas (Brugiavini et al. 2017; Gori and Fernandez 2015). For example, a person with two limitations in ADL and three in iADL—a common profile for the European older population—may be eligible for home care in Belgium but not in Germany, depending on the specific combination of iADL and ADL limitations (Table 1). As summarized in Table 1, Profiles A and B are eligible for home care under the Belgian legislation, while profiles C and D are eligible under the German rules (Carrino, Orso, and Pasini 2018). Moreover, the Belgian's APA gives the same weight to ADL and iADL, whereas Germany assigns outcome‐specific need‐of‐care allotments (with ADLs having higher weights) and requires that ADL‐difficulties account for a minimum of 45 min‐of‐care (this explains why profiles A and B in Table 1 are not eligible in Germany). Conversely, bathing and eating have the highest weights in the German but not in the Belgian rules, hence the outcome of profile D.

**TABLE 1:** Clinical profiles evaluated under the eligibility rules of Belgium and Germany.

Profile A	Profile B	Profile C	Profile D
Limited in 2 ADL, 3 iADL	Limited in 3 ADL, 3 iADL	Limited in 2 ADL, 3 iADL	Limited in 3 ADL, 3 iADL
**Age**: 74	Age: 85	Age: 74	Age: 84
**Limitations in ADL**: Dressing, bathing	**Limitations in ADL**: Dressing, bathing, transferring	**Limitations in ADL**: Incontinence, bathing	**Limitations in ADL**: Bathing, eating, using WC
**Limitations in iADL**: Outdoor mobility, using the telephone, managing money	**Limitations in iADL**: Shopping for groceries, meal preparation, houseworks	**Limitations in iADL**: Outdoor mobility, shopping for groceries, houseworks	**Limitations in iADL**: Shopping for groceries, houseworks, managing money
**Cognitive limitations**: Yes	**Cognitive limitations**: No	**Cognitive limitations**: No	**Cognitive limitations**: No
**Eligibility status**: ELIGIBLE IN BELGIUM	**Eligibility status**: ELIGIBLE IN BELGIUM	**Eligibility status**: ELIGIBLE IN GERMANY	**Eligibility status**: ELIGIBLE IN GERMANY

The exogeneity of the instrument stems from its legislation‐based nature and is contingent upon health status and functioning: the way a legislation evaluates a specific combination of health‐outcomes should not directly affect a respondent's psychological well‐being, except through its impact on the probability of receiving home‐based care. Importantly, depression is either absent or assigned a very limited weight in the eligibility algorithms (Brugiavini et al. 2017). As worse health and function increases eligibility, our main specification controls for an extensive set of individual health conditions comparable to those included in the legislations (number of ADL and iADL limitations, having any mobility limitation or low cognitive function), besides other covariates (Section 4.1). Our results are also robust to including a full set of dummies for each ADL and iADL limitation (Section 5.1). This ensures that the eligibility index only captures the exogenous increase in the individual probability of receiving care, determined exogenously by the functional form of the eligibility algorithm. Notice that any bias arising from unobserved health heterogeneity would bias IV estimates downwards, as poorer physical health is associated with higher probability of both being eligible for LTC and having depression symptoms (see Sections 2 and 3). Thus, any unobserved health heterogeneity would suggest that our estimates represent a lower bound estimate of the impact of receiving LTC on mental health.

To inform on the potential exogeneity of the instrument, we test whether the LTC eligibility index has any predictive power on unrelated individual characteristics, such as number of children, marital status, education, income, wealth, and residential area. We estimate the association between individuals' LTC eligibility status and a set of outcomes Y, controlling for individual health characteristics (HS) and regional dummies (*R*), through the following model (2) and discuss its findings in Section 5.1.

(2) $$Y_{i,r}=\gamma_{0}+\gamma_{1}{\text{eligible}}_{i,r}+\gamma_{2}^{\prime }{\text{HS}}_{i,r}+\gamma_{3}{R_{r}+\varepsilon }_{i,r}$$

Finally, bias may arise if medical evaluators who take the decision on eligibility deviate from the strict application of laws and guidelines. We discuss the impact of this potential bias in Section 4.3.

An important point to note is that both eligible individuals (our treatment group) and non‐eligible individuals (our control group) have in theory the choice of a mix bundle of informal care and privately purchased formal care. The difference between eligible (treatment) and non‐eligible (control) participants is that the treatment group is eligible to additionally receive public formal care support. We empirically show that the treatment group indeed receives more formal care than the control group (theory predicts they would reduce private care because of enhanced public care provision). Individuals who are not eligible for public LTC but who have limitations, do not receive public LTC because they do not meet the requirements to do so in their country of residence (even if they may have a combination of physical limitations that are similar to those faced by those who are eligible, but not exactly aligned with the eligibility criteria). This control group, therefore, is a combination of several individuals: a large majority of them are likely to receive no or little formal care, as costs of long‐term care are generally prohibitive for a large fraction of the population in the countries assessed. Instead, many of them rely on informal care to meet their LTC needs. Third, for certain individuals, particularly those with higher income, it may be possible to purchase formal care support in the private market. The specific combination of these three options depends on individual circumstances and country of residence.

#### 4.2.2 Instrumental Variables for Informal Care

Formal and informal care are jointly determined when deciding the amount of care an individual needs. Therefore, we include information on informal care use in our empirical model. We are therefore required to identify an instrumental variable for informal care that allows us to estimate a causal effect on depression that is, not influenced by the availability of formal LTC.

To instrument informal care, prior studies have used information on children characteristics—for example, the number of children, the fraction or the number of daughters, the existence of children with no partners and/or no children—as these are assumed to affect the amount of informal care demanded by the dependent person exogenously, for example, unrelated to her health characteristics (Barnay and Juin 2016; Bolin, Lindgren, and Lundborg 2008; Bonsang 2009; Kolodziej, Coe, and Van Houtven 2022; Urwin, Lau, and Mason 2019; Van Houtven and Norton 2004). The theoretical assumptions are that (i) a larger number of offspring results in a broader support network of caregivers for older adults; and that (ii) daughters are more willing to provide care than sons (Grigoryeva 2017).^3^ Based on these assumptions, we select number of children and fraction of daughters as instruments for informal care, and we discuss their econometric properties hereafter. We restrict our sample to individuals with children and focus on a measure of informal care from respondents' offspring. The limitations arising from this selection are discussed in the final section of the paper.

The exclusion restriction requires that, among people having at least one child, the number and gender composition of the offspring influence the parent's psychological well‐being only through informal care support and not directly. The evidence on the direct effect of number of children on parents' depression is inconclusive. As noted by Kolodziej, Coe, and Van Houtven (2022), there is no evidence that family structure, for example, size, birth order, and gender mix, systematically affects the mental health of the adult parent. A European study employing the same dataset as in our study finds that the number of children does not directly affect a person's psychological well‐being (Kruk and Reinhold 2014). Conversely, a study from the US finds beneficial effects of a larger offspring for white mothers only (van den Broek 2020; van den Broek and Tosi 2020). These studies argue that the offspring size mainly affect parents' mental health through its impact on caregiving support (which would satisfy the exclusion restriction in our model), however, they do not test this assumption directly. As for gender composition of the offspring, while it is possible that mothers prefer daughters as caregivers (Suitor and Pillemer 2006), there is no evidence that the number of daughters directly affect parent's mental health (van den Broek 2020).

However, there might still be concerns that the number of children could affect the mental health of the parent through alternative channels other than informal caregiving, for example, through partially unobserved confounders such as family socioeconomic status and cultural norms. In order to address such concerns, we have included two alternative identification strategies in the robustness section. First, we implemented models that use only the fraction of daughters as instrument for informal care: this instrument is more likely to satisfy the exclusion restriction as it captures a children characteristic which is less likely to be the result of a choice by the respondent than the offspring's size. Second, we further test the robustness of our estimates of the impact of formal home‐based care –our main variable of interest—to excluding informal care as a covariate in models, and replacing it with children's characteristics.^4^

Finally, we note that, in light of the potential criticisms to the instrumental variables used for informal care, the interpretation of the impact of informal care use on depression and wellbeing must be taken with caution. The focus of this paper lies on the impact of formal care on health and well‐being. As shown in the robustness section, such impact is robust to using different instruments for informal care.

### 4.3 Data

Data comes from waves 1 (2004), 2 (2007), 5 (2013) 6 (2015) and 7 (2017) of the Survey of Health, Aging and Retirement in Europe (SHARE), a large cohort study representative of populations aged 50 and older in 27 European countries (Börsch‐Supan et al. 2013). We do not use Waves 3 and 4 as they lack information on formal‐care use. Our country selection includes Belgium, France, Germany and Spain (see Section 4.2).^5^

We focused on non‐institutionalized respondents aged 60 and older. This cutoff is based on the fact that the age of 60 is the lowest limit included in the legislations that have age restrictions, as described in Section 4.2. Moreover, while loss of autonomy and LTC use is higher at older ages, our data indicates that a non‐trivial proportion of SHARE respondents have substantial loss of autonomy and are receiving LTC while aged 60 to 64. In particular, around 2.8% of respondents aged 60 to 64 in our analytical sample are eligible to receive LTC support, and 2.7% are receiving formal LTC support; while among those aged 65+, 8.5% are eligible to public LTC support, and 8.6% are receiving formal LTC support. In the online Supporting Information S1: Appendix 3 we present sensitivity analyses for the sample restricted to people aged 65 years and older, and 70 years and older.

Given that we instrument informal care use based on the number of children and fraction of daughters, as is commonly done in this literature (Section 4.2), our sample is restricted to older adults with children.

#### 4.3.1 Mental Health and Well‐Being

Depressive symptoms are assessed using the continuous Euro‐D score, which ranges from 0 to 12 and was specifically designed and validated for older Europeans (Prince et al. 1999). It includes items on sadness, pessimism, suicidality, guilt, sleep, interest, irritability, appetite, fatigue, concentration, enjoyment, and tearfulness, each scored either 0 (symptom not present) or 1 (symptom present, see Supporting Information S1: Appendix 1). The Euro‐D score effectively predicts clinically significant depression, with an optimal cutoff point of 4 or above (Castro‐Costa et al. 2007).

We also examine impacts on well‐being using a continuous index of Quality of Life (QoL) in older age, the CASP‐12, and its four sub‐components. CASP‐12 is a reduced version of the widely‐used CASP‐19 scale (Borrat‐Besson, Ryser, and Gonçalves 2015; Hyde et al. 2003), which reflects four dimensions of QoL: control, autonomy, self‐realization, and pleasure (see Supporting Information S1: Appendix 1). As no clinically validated threshold has been proposed, we generate a binary indicator for being above or below the average CASP score in our sample (37.9). We employ the official CASP score as generated by the SHARE team. Due to partial missing responses, the CASP score is available for a slightly smaller sample than the EURO‐D, accounting to 15,468 individuals and 29,703 observations.

We also assess the impact of formal care on feelings of loneliness, measured through the three‐item University of California, Los Angeles (UCLA) Loneliness Scale. The scale captures feelings of (1) lack of companionship, (2) being left out, and (3) being isolated from others. A threshold of 6 (out of a maximum of 9) defines the caseness of loneliness (Russell, Peplau, and Ferguson 1978). The loneliness variable is generated directly by the SHARE team, and is available from wave 5 onwards, for 12,651 individuals and 23,246 observations in our sample.

#### 4.3.2 Formal and Informal Care Utilization

SHARE participants are asked about the utilization of formal and informal domiciliary help over the previous 12 months in relation to health problems. Based on prior studies (Carrino, Orso, and Pasini 2018), we define formal care as the receipt of professional or paid personal/nursing care, or meals on wheels (MOW) services (while MOW are typically supplied by formal care providers, results are robust to excluding them from the definition of formal care).

SHARE assesses informal care received from family, friends and neighbors. Respondents are specifically asked to identify their caregivers, enabling a detailed understanding of their support network. They are subsequently asked to report the frequency of care received by adult children. Our analysis focuses on informal care provided by children, as detailed in Section 4.2.

#### 4.3.3 Sociodemographic, Regional, and Health Controls

We control for the following variables: age, age squared, gender, marital status/cohabitation, highest education attainment (ISCED), residential area (urban vs. rural), household income and wealth quintiles, NUTS1 region, wave, self‐reported health, ADL and iADL lost, mobility limitations, and cognitive function. We employ the imputed measures of income and wealth produced by the SHARE team using multiple imputation, as described in De Luca, Celidoni, and Trevisan (2015). We employ SHARE generated variables on health (e.g., ADL, IADL, mobility loss, memory scores, orientation score) and sociodemographic characteristics (e.g., education), to minimize discrepancies and missing values.

We note that individuals are less likely to provide a valid answer to the cognitive tests in SHARE (orientation questions, memory test) and to other questions (Schneider et al. 2024). Therefore, the SHARE sample is less likely to represent the population with severe cognitive decline. This implies that our results are not likely to capture the full effect of receiving publicly subsidized long‐term care among people with severe cognitive limitations.

Details on the coding of these variables are reported in Table 2, which summarizes descriptive statistics for our main sample.

**TABLE 2:** Descriptive statistics.

	All sample		Belgium		France		Germany		Spain	
	Mean	SD	Mean	SD	Mean	SD	Mean	SD	Mean	SD
Age	71.056	7.876	71.058	7.926	71.291	8.199	70.148	7.237	72.329	8.135
Being female (y/n)	0.545	0.498	0.543	0.498	0.571	0.495	0.513	0.500	0.565	0.496
Living in couple (y/n)	0.730	0.444	0.715	0.451	0.658	0.474	0.784	0.412	0.788	0.409
Low education (y/n)^a^	0.452	0.498	0.477	0.499	0.505	0.500	0.153	0.360	0.862	0.345
Mid education (y/n)^a^	0.318	0.466	0.244	0.430	0.300	0.458	0.556	0.497	0.065	0.246
High education (y/n)^a^	0.230	0.421	0.279	0.448	0.195	0.396	0.290	0.454	0.074	0.262
Living in rural area (y/n)	0.299	0.458	0.249	0.433	0.405	0.491	0.384	0.486	0.064	0.244
EURO‐D score	2.508	2.241	2.426	2.161	2.822	2.253	2.114	1.943	2.876	2.719
EURO‐D caseness	0.282	0.450	0.269	0.444	0.337	0.473	0.214	0.410	0.343	0.475
CASP score	37.716	6.103	38.075	5.957	37.575	6.117	39.002	5.495	34.970	6.486
CASP control	8.669	2.256	8.598	2.215	8.484	2.268	9.134	2.097	8.335	2.429
CASP autonomy	9.264	1.886	9.556	1.862	9.315	1.852	9.426	1.784	8.438	1.901
CASP pleasure	10.452	1.826	10.297	1.933	10.403	1.829	11.041	1.398	9.880	1.963
CASP self‐realization	9.379	2.199	9.708	2.099	9.523	2.158	9.565	2.073	8.317	2.295
UCLA binary loneliness	0.112	0.315	0.129	0.335	0.123	0.328	0.073	0.259	0.127	0.333
Bad subjective health	0.097	0.296	0.055	0.229	0.112	0.315	0.098	0.297	0.159	0.366
# ADL limitation^b^	0.268	0.835	0.281	0.794	0.250	0.775	0.227	0.795	0.345	1.054
# iADL limitation^c^	0.460	1.212	0.490	1.191	0.430	1.122	0.333	1.036	0.684	1.606
Any mobility limitation (y/n)^d^	0.561	0.496	0.574	0.494	0.562	0.496	0.545	0.498	0.564	0.496
Low cognitive function (y/n)^e^	0.070	0.256	0.069	0.253	0.060	0.237	0.051	0.220	0.129	0.335
Gets formal home care (y/n)	0.076	0.264	0.098	0.297	0.096	0.294	0.053	0.225	0.035	0.184
Gets care from children (y/n)	0.101	0.302	0.105	0.307	0.091	0.288	0.101	0.302	0.109	0.311
Observations	33,178		10,544		8464		9195		4975	
Respondents	16,627		4909		4190		5059		2469	

*Note:* The sample includes individuals aged 60+, having children, in SHARE waves 1, 2, 5–7 in Belgium, France, Germany and Spain.

^a^ Coded using ISCED 1997 codes: up to lower secondary (ISCED 0–2); upper secondary (3–4); tertiary (5–6).

^b^ Min = 0, max = 6 (e.g., dressing, walking across a room, bathing).

^c^ Min = 0, max = 7 (e.g., preparing a hot meal, shopping for groceries, making telephone calls).

^d^ Min = 0, max = 10 (e.g., walking 100 m, sitting for 2 h, getting up from a chair).

^e^ = 1 if respondent has either a low memory score (i.e., fewer than 8 out of 20 words recalled) or a low time orientation score (i.e., two or more mistakes in identifying day of the week, date, month and year).

#### 4.3.4 Descriptive Statistics

After discarding observations with missing values in any variable of interest, our main sample includes 16,975 individuals, and 33,178 observations for the EURO‐D depression outcomes; 15,468 individuals and 29,703 observations for the CASP Quality of Life outcomes; and 12,651 individuals and 23,246 observations for the loneliness outcome.

The average age is 74. 9.2% of respondents report receiving formal home care, while around 12.4% receive informal home care from their children.

#### 4.3.5 LTC‐Eligibility

Table 3 provides details of the vulnerability dimensions used in the assessment of need in each country to determine eligibility status, all of which are available in SHARE. The ADL group resembles the taxonomy introduced by Katz et al. (1970), whereas the non‐ADL is a residual set which includes iADL and cognitive/behavioral limitations. Each LTC program assesses a different sub‐set of these outcomes and builds an “eligibility index” for each applicant, through different non‐linear and non‐additive algorithms. Functional and cognitive limitations are critical factors in determining eligibility status, although their influence varies across different programs. In contrast, mental disorders are either excluded from consideration or given minimal weight (Brugiavini et al. 2017).

**TABLE 3:** Summary of vulnerability outcomes included in need‐assessment scales.

ADL	Non ADL
Bathing & hygiene	Communication
Dressing	Shopping for groceries/medicines
Using the toilet	Cooking
Transferring	Housework
Continence	Moving outdoor
Feeding	Responsibility for own medications
Moving indoor	Cognitive impairment
	Behavioral/mental disorders
	Other mobility limitations

*Note:* The underlined tasks do not belong to the Katz's ADL scale, but are treated as basic activities of daily livings in the LTC regulations that include them. Additional mobility limitations include, for example, crouching and walking downstairs.

Using self‐reported information, we build a health profile for each SHARE respondent (Brugiavini et al. 2017), and contrast it with the requirements of the prevailing legislation in the respondent's country or region of residence, to establish whether they would in theory be eligible to receive home‐based care.^6^

Medical evaluators who take the decision on eligibility may deviate from the strict application of laws and guidelines. In line with previous research (Carrino, Orso, and Pasini 2018), we assume that those deviations are not systematic. While some studies have found that evaluators do sometimes deviate systematically from the law (Maestas, Mullen, and Strand 2013), there are several reasons why this is less of a concern for our study. First, we focus on individuals from different countries, each of which has different evaluation procedures, making it unlikely that there is a systematic deviation common to all countries. In addition to individual covariates, all our specifications include a full set of regional dummies to account for unobservable effects common to individuals from the same region, including systematic deviation of medical evaluators facing the region‐specific LTC regulation. Our assumption is that any remaining unobservable variation in the deviations from the strict application of diverse LTC legislations can be safely assumed to be idiosyncratic.

Table 4 reports descriptive statistics on home‐based care utilization for the eligible population (7.5% of the sample) and compares it with the population with activity restrictions (both ADL and iADL), as well as with the whole sample.

**TABLE 4:** Descriptive statistics for the eligible population.

	All sample		Population eligible to LTC		Population with activity restrictions	
	Mean	SD	Mean	SD	Mean	SD
Age	71.056	7.876	78.088	8.768	75.414	8.763
Being female (y/n)	0.545	0.498	0.599	0.490	0.633	0.482
Living in couple (y/n)	0.730	0.444	0.540	0.498	0.617	0.486
Low education (y/n)^a^	0.452	0.498	0.666	0.472	0.605	0.489
Mid education (y/n)^a^	0.318	0.466	0.238	0.426	0.254	0.436
High education (y/n)^a^	0.230	0.421	0.096	0.295	0.141	0.348
Living in rural area (y/n)	0.299	0.458	0.337	0.473	0.303	0.459
EURO‐D score	2.508	2.241	4.425	2.582	3.865	2.488
EURO‐D caseness	0.282	0.450	0.605	0.489	0.510	0.500
Bad subjective health	0.097	0.296	0.411	0.492	0.272	0.445
# ADL limitation^b^	0.268	0.835	2.392	1.825	1.109	1.426
# iADL limitation^c^	0.460	1.212	3.184	2.464	1.918	1.885
Any mobility limitation (y/n)^d^	0.561	0.496	0.930	0.255	0.931	0.253
Low cognitive function (y/n)^e^	0.070	0.256	0.428	0.495	0.160	0.366
Formal home care (y/n)	0.076	0.264	0.428	0.495	0.219	0.414
Care from children (y/n)	0.101	0.302	0.358	0.480	0.276	0.447
Observations	33,178		2481		8438	

*Note:* The sample includes individuals aged 60+, having children, in SHARE waves 1, 2, 5–7 in Belgium, France, Germany and Spain.

Activity restriction is defined as having at least 1 ADL and iADL limitations.

^a^ Coded using ISCED 1997 codes: up to lower secondary (ISCED 0–2); upper secondary (3–4); tertiary (5–6).

^b^ Min = 0, max = 6 (e.g., dressing, walking across a room, bathing).

^c^ Min = 0, max = 7 (e.g., preparing a hot meal, shopping for groceries, making telephone calls).

^d^ Min = 0, max = 10 (e.g., walking 100 m, sitting for 2 h, getting up from a chair).

^e^ = 1 if respondent has either a low memory score (i.e., fewer than 8 out of 20 words recalled) or a low time orientation score (i.e., two or more mistakes in identifying day of the week, date, month and year).

The “eligible” subsample is notably different from the generically “impaired” population in terms of (i) higher risk of depression (60% vs. 51%); (ii) worse self‐reported health; (iii) higher number of ADL and IADL limitation, and; (iv) worse cognitive function. The “eligible” subsample has also higher prevalence of formal care use (43%), than the activity‐restriction sample (22%), and the total sample (8%). Finally, among the eligible population, the frequency of formal care use is similar to that of receiving informal care, while informal care is more common than formal care in the other two (sub)samples.

**FIGURE 1:**
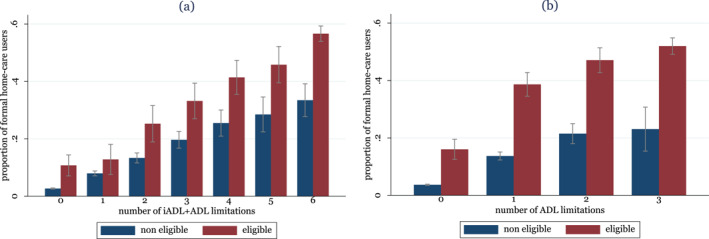
Proportion of respondents receiving formal home care, by number of ADL/iADL limitations and by LTC eligibility status (non eligible vs eligible to local public programmes of LTC). The sample includes individuals aged 60+, having children, in SHARE waves 1, 2, 5–7 in Belgium, France, Germany and Spain.

Figure 1 shows that, as expected, the probability of receiving formal care increases with the number of functional limitations. Among respondents with the same degree of activity restriction (measured with the number of limitations in both ADL and iADL in panel a or just in ADL in panel b), the probability of receiving formal care is systematically higher in the eligible group than in the non‐eligible group. To illustrate, among those who have 3 ADL/IADL limitations but are not eligible for home‐based care, 20% receive help as compared to around 35% of those with same number of limitations who are eligible.

## 5 Results

### 5.1 First Stage Results

Table 5 reports the coefficients for the first stage regressions for formal home‐based care (column 1) and for informal care (column 2). For formal home‐based care, the instrument is the eligibility criteria in the county or region of residence as set out in local legislation, while for informal care we use the number of children and the proportion of daughters. The instruments significantly predict the use of long‐term care. The LTC eligibility index is a strong predictor of formal‐care use (*F*‐statistic = 31.9), above and beyond the effect of sociodemographic and health characteristics. In line with previous evidence (Carrino, Orso, and Pasini 2018), being eligible to receive formal care according to the legislation in the country or region of residence increases the probability of receiving home‐based care by 9.9 percentage points, relative to individuals with similar levels of impairment and demographics but who are not eligible by virtue of the eligibility criteria set out in the legislation. Likewise, a larger number of children (*F*‐statistic = 27.9) and a larger fraction of daughters (*F*‐statistic = 13) have a positive and significant effect on the probability of receiving informal care from children (Bonsang 2009). Overall, diagnostic tests confirm that instrumental variables are relevant and not weak.

**TABLE 5:** Effect of receiving home care on depression.

	(1) Any formal home care	(2) Any informal care (from children)	(3) EURO‐D OLS	(4) EURO‐D IV
Eligible to home care	0.099***	0.012	—	—
	(0.017)	(0.015)		
Fraction of daughters	0.000	0.001***	—	—
	(0.001)	(0.000)		
Number of children	0.000	0.008***	—	—
	(0.001)	(0.001)		
Any formal home care	—	—	0.154***	−2.615***
			(0.042)	(0.944)
Any informal care (children)	—	—	0.130**	−1.150
			(0.049)	(1.310)
Age	−0.037***	−0.034***	0.123***	−0.071
	(0.005)	(0.005)	(0.045)	(0.045)
Age^2^	0.001***	0.001***	−0.001***	0.000
	(0.000)	(0.000)	(0.000)	(0.000)
Female	−0.001	0.022***	0.635***	0.612***
	(0.003)	(0.003)	(0.050)	(0.049)
In couple	−0.033***	−0.094***	−0.153***	−0.279**
	(0.004)	(0.007)	(0.034)	(0.130)
Low educ (ref.: High)	0.005	0.005	0.287***	0.300***
	(0.005)	(0.005)	(0.045)	(0.042)
Mid educ (ref.: High)	0.002	0.007	0.061*	0.065
	(0.004)	(0.006)	(0.036)	(0.044)
Bad subject. health	0.029***	0.025***	1.437***	1.521***
	(0.007)	(0.009)	(0.058)	(0.066)
# adl	0.059**	0.032**	0.292***	0.481**
	(0.026)	(0.015)	(0.101)	(0.220)
# iadl	0.043***	0.048***	0.236***	0.355***
	(0.005)	(0.005)	(0.021)	(0.067)
Any mobility limit.	0.007***	0.089***	0.672***	0.676***
	(0.002)	(0.005)	(0.037)	(0.122)
Low cognitive health	−0.006	0.011	0.422***	0.471***
	(0.008)	(0.008)	(0.061)	(0.062)
#adl * mobility limit.	−0.030	−0.031*	−0.165	−0.230
	(0.026)	(0.016)	(0.130)	(0.194)
*F‐test*—Eligible for home care	31.9	0.68		
*F‐test*—Fraction of daughters	2.1	13		
*F‐test*—Number of children	0.14	27.9		
Sanderson‐Windmeijer *F*‐test of excluded instruments	16.4***	28.16***		
*N*	33,178	33,178	33,178	33,178
Sample average	0.092	0.13	2.508	2.508

*Note:* We report results for the first‐stage (columns 1, 2) and the OLS and IV models for EURO‐D scores (columns 3, 4), estimated using model (1). Sample: individuals aged 60+, having children, in SHARE waves 1, 2, 5–7 in Belgium, France, Germany and Spain. Further controls include: household income quintiles, area of living (urban vs. rural), dummies for waves and NUTS1 regions. Standard errors clustered by NUTS1 regions (57).

Statistical significance: *p < 0.1, **p < 0.05, ***p < 0.01.

#### 5.1.1 LTC Use and Depression Score

Our main results for the second stage are reported in Table 5. We evaluate the magnitude and clinical relevance of the results by computing the “effect size” (ES), that is, the ratio between an estimated coefficient and the standard deviation of the outcome (J. Cohen 2013), with small, medium and large ES corresponding to 0.2 SD, 0.5 SD and 0.8 SD, respectively.

Column 3 in Table 5 reports the results for the baseline model that does not account for endogeneity of care use: receiving formal care is associated with a 0.15 higher EURO‐D depression scores, a significant but small coefficient given that the average EURO‐D score is 2.5 with a standard deviation of 2.2. This estimate is likely to be biased by selection and unobserved heterogeneity. We therefore turn to our main instrumental variable specific in column 4, which shows that the use of formal home‐based care (as a result of being eligible according to local legislation) significantly reduces depression scores by 2.6 points, which constitutes a large Cohen *d* effect. By contrast, although coefficients also turn negative in the IV model, the effect of receiving informal care on depression scores is not statistically significant. Coefficients for control variables are in line with previous findings: depression scores are higher for women, people living alone, those with lower levels of education, as well as for people with more functional limitations (ADL, iADL, mobility), cognitive impairments, or those who report worse self‐assessed health.

The substantial change of sign and size in the coefficient of interest between the OLS and the instrumented model can be explained by two characteristics of the IV approach. First, the implementation of the instrumental variable allows us to correct for endogeneity bias in the OLS coefficients; as explained in Section 4.2 and in previous studies, such bias is likely to be positive, that is, over‐estimating a positive association between care use and depression. Second, the IV model essentially estimates the impact of receiving any formal care by comparing the depression scores among the population of compliers, that is, respondents who start receiving care because they are eligible for it, and a “counterfactual” group of respondents with similar health limitations, but who are not receiving formal care because they are not eligible.^7^ It would be ideal to exploit information on hours of care received by respondents, to compute a more precise estimate of the unitary impact of care hours on depression. However, information on hours of care received is only available for the first two waves of SHARE.

#### 5.1.2 Risk of Clinical Depression

To evaluate the clinical relevance of our findings, we estimate how formal care use impacts the probability of possible clinical depression, employing the EURO‐D caseness index (Table 6, column 1) (Castro‐Costa et al. 2007). Our IV‐estimates suggest that formal care utilization leads to a 13.3 percentage point reduction in the probability of possible clinical depression. This is a substantial effect, when compared to an average prevalence of 28.2% in the sample. Receiving informal care reduces the probability of depression by 7.7 percentage points.

**TABLE 6:** Effect of receiving home care on depression.

IV estimates	(1) EURO‐D caseness	(2) CASP score	(3) CASP control	(4) CASP autonomy	(5) CASP self‐realization	(6) CASP pleasure	(7) CASP median caseness	(8) Loneliness caseness
Any formal home care	−0.133***	2.084	1.637**	0.875	−0.472	0.197	0.147***	−0.067***
	(0.020)	(2.246)	(0.773)	(1.031)	(0.824)	(0.720)	(0.033)	(0.013)
Any informal care (children)	−0.077***	1.132	−0.024	−5.879***	2.168	2.350***	0.160***	−0.064***
	(0.025)	(4.437)	(1.290)	(1.842)	(1.515)	(0.992)	(0.034)	(0.023)
SW *F*‐test instruments for FC equation	15.6	22.9	22.9	22.9	22.9	22.9	22.9	26.75
SW *F*‐test instruments for IC equation	28.6	17.3	17.3	17.3	17.3	17.3	17.3	11.27
*N*	33,178	29,703	29,703	29,703	29,703	29,703	29,703	23,246
Sample average	0.282	37.7	8.67	9.26	9.38	10.45	0.5	0.112

*Note:* Sample: individuals aged 60+, with 1+children, in SHARE waves 1, 2, 5–7 in Belgium, France, Germany and Spain. Controls: age (quadratic), gender, living arrangements, education, living area, self‐reported health, ADL limitations, iADL limitations, mobility limitations, cognitive health, fixed‐effects for household income (quintiles), waves and NUTS‐1 regions. Standard errors are clustered by NUTS‐1 regions (57).

Statistical significance: *p < 0.1, **p < 0.05, ***p < 0.01.

The magnitude of our findings is relevant when compared to recent studies which used the same dataset (SHARE). The impact of formal care on depression is comparable, in absolute terms, to the depression effect of being married (−13.8 p.p.) or feeling limited in functional activities (+19.7 p.p.) (Avendano et al. 2015); while it is larger, in absolute terms, than the impact of providing informal care on a caregiver's depression risk (+7 p.p., +12 p.p for intense caregiving) estimated by Brenna and Di Novi (2016). Our estimates for home‐based care on depression scores are also comparable to the effect of retirement on depression scores, as reported by Kolodziej and García‐Gómez (2019) (−16.4 p.p.); and the effect of life‐course determinants such as education and maternity leave coverage: an extra year of compulsory education lowers older age depression risk by 6.5 p.p. (Crespo, López‐Noval, and Mira 2014), while being covered by a comprehensive maternity leave reduces it by 10 p.p. (Avendano et al. 2015).

#### 5.1.3 Quality of Life

We further examine the impact of home‐based care use on Quality of life (QOL), measured with the CASP scale (Borrat‐Besson, Ryser, and Gonçalves 2015). We present results for the overall CASP score, for each score dimension (control, autonomy, self‐realization, pleasure), and for a binary indicator capturing whether respondents are above the median levels of CASP score. Although we find no significant effect on overall CASP scores, our IV‐estimation suggests that formal care use has a large positive effect on the control dimension (column 3), that is, on the self‐perception of being able to shape life through one's own behavior. This supports the theoretical prediction that formal care restores capability to achieve *functionings* and hence improves QOL. Results on other CASP dimensions suggest improvements in QOL, but estimates are not statistically significant (columns 2, 4–6). When focusing on the binary CASP indicator, we predict that formal care use increases by 14.7 percentage points the likelihood of reporting an above‐median QOL (7), suggesting that the net‐effect of care use is large enough to improve the relative position of respondents in the QOL distribution.

Results for informal care suggest that receiving care from children does not improve overall CASP scores. This may be the result of conflicting effects on eudemonic well‐being (pleasure and control‐autonomy), which captures the possibility to flourish in life. We find that informal care increases pleasure scores (6), which measures the aspects of living (pursuit of enjoyable activities and the fulfillment of oneself) that contribute to increase happiness. By contrast, informal care decrease the autonomy scores, which measures the extent to which participants feel that they can make their own decisions. These contradictory effects suggest that informal care may contribute to higher enjoyment of life at the cost of a loss of autonomy, potentially due to dependence on adult children. Overall, however, informal care use increases the probability of reporting a higher than median CASP scores (7).

#### 5.1.4 Loneliness

A potential mechanism by which formal home‐based care impacts depression might be by reducing feelings of loneliness. Employing the UCLA binary Loneliness index (available in waves 5–7), we find that receiving formal home‐based care reduces the probability of reporting feelings of loneliness by 6.7 percentage points. This corresponds to a substantial reduction, compared to the average prevalence of loneliness (11.2%). We find that receiving informal care leads to a similar reduction in the probability of reporting feelings of loneliness. This supports theoretical predictions that formal care can improve quality of life and mental health by reducing feelings of loneliness.

#### 5.1.5 Further Mechanisms Analysis

In Supporting Information S1: Appendix 4, we further explored potential mechanisms through which access to publicly subsidized formal home care can improve mental health and quality of life, with particular reference to hospitalization and home adaptations. We find some weak evidence that access to formal LTC leads to reduced hospitalization, and that it increases the probability of implementing home adjustments, which may lead to improved health and quality of life.

### 5.2 Robustness Checks

We conducted a series of comprehensive sensitivity analyses, detailed in Supporting Information S1: Appendix 3.

As a summary, we report that (i) we found no evidence of heterogeneity by socioeconomic status in the effect of care on depression and quality of life (Supporting Information S1: Table 12); (ii) the eligibility index satisfies the placebo/falsification test (Supporting Information S1: Table 13); (iii) the results are robust to including a larger set of health controls (Supporting Information S1: Table 14); (iv) results are confirmed in a sample that excludes the oldest old (90+ years) (Supporting Information S1: Table 14) as well as to; (v) restricting the sample to respondents aged 65 or more, and 70 or more (Supporting Information S1: Tables 15 and 16). Furthermore, we verified the robustness of our findings regarding the impact of formal care utilization on health and well‐being to various strategies addressing the endogeneity of informal care. Specifically, we employed the fraction of daughters as an instrument, without considering the total number of children (Supporting Information S1: Tables 17 and 18).^8^ Subsequently, we tested an alternative model that omits informal care use as an independent variable, replacing it with characteristics of children, including both the number of children and the fraction of daughters (Supporting Information S1: Table 19). Our analyses indicate that our main results were robust and did not exhibit significant changes across these models.

Our study has provided an estimate on the average effect of LTC in the four countries considered. In Supporting Information S1: Appendix 3, we show that our results do not change systematically when excluding any country from the sample (Supporting Information S1: Figure 2 in Appendix 3). While it would be interesting to study the specific impact of formal care in different institutional contexts, we cannot perform country‐specific analyses due to insufficient data to support the identification strategy at country level. For this reason, while we find no strong evidence to believe that the average effect of LTC we observe across all countries is driven by any particular country, further research is needed to provide more detailed comparisons across LTC systems.

## 6 Discussion

Our paper provides new estimates of the impact of publicly subsidized home‐based care on the mental health and well‐being of older people in Europe. We use a novel instrumental variable approach that exploits geographical differences in legislation, and that allows us to overcome some of the challenges in estimating the impact of long‐term care use on the health and well‐being of older people.

Our study shows that the net effect of home‐based LTC on mental health and quality of life is positive and relevant: older people who receive formal home‐based care by virtue of their eligibility for public programmes have better mental health and quality of life than those who are not covered by formal home care services. In particular, our findings suggest that using home‐based care leads to a significant and large reduction in depressive symptom scores, and the probability of clinically meaningful depression. In addition, the use of home‐based formal care increases quality of life as measured by the CASP scale, particularly by increasing feelings of control over life. We show that a potential mechanism involves the impact of home‐based care on loneliness: we estimate that receiving formal home‐based care reduces the risk of loneliness by 6.7 percentage points. Potential alternative mechanisms include reduced hospitalization and improved housing conditions. Our results provide evidence that formal home‐based care enhances mental well‐being and quality of life, potentially through increased feelings of control over life and reduced feelings of loneliness (Berkman et al. 2000; Hu and Wang 2019; Stabile, Laporte, and Coyte 2006; Thomas, Akobundu, and Dosa 2016; Wolff and Agree 2004).

Estimates of the “excess cost” of depressive disorders in older age (e.g., drugs, nursing and social care) shed some light on the societal welfare effects of home‐based care (König, König, and Konnopka 2020). Using data for the UK, McCrone et al. (2008) estimates that the excess costs of depression are $3225 per year for an individual aged 65–74 in 2006. In Germany, Bock et al. (2016) estimates similar excess costs of depression at $2840 per year for an older adult aged 75+ in 2012.^9^

Our results provide strong evidence that public investments in home‐based care may be justified in terms of improved mental health and well‐being outcomes for older people. Our findings support the implementation of more inclusive eligibility criteria for home‐based LTC and suggest that budget cuts to LTC services should take into account the potential welfare losses for older individuals. It is also important to consider the distributional impacts of LTC: given that the costs of care can exceed median income and last for several years, home care offers a social protection against the financial risks associated with functional decline and the need for LTC, thus also contributing to reduce poverty (Hashiguchi and Llena‐Nozal 2020). By also improving mental health and well‐being, formal home‐based care can contribute to reduce inequalities in health in older adults (Steptoe, Deaton, and Stone 2015), while reducing pressure on healthcare costs and informal care support.

Our results are also relevant for current debates on how to sustain quantity and quality of the future workforce in LTC (OECD 2020). The well‐being gains generated by LTC use are only possible through an adequate LTC workforce. Most countries with advanced LTC systems face workforce shortages and challenges in recruiting high skilled workers, often due to poor working conditions and low pay. To meet these challenges, governments are implementing programmes of professionalization of the LTC workforce, for example, through formal registration, enhanced education, improvements in working pay and terms. Our results suggest that these programmes may pay off in terms of improved well‐being of frail older individuals.

Several limitations in our analysis should be considered.

A first set of limitations has to do with the quality of data on care use, and on the binary nature of the eligibility variable. In this paper, we focus on the extensive margin of care use, rather than on the intensive margin, given the lack of information on formal care hours in the SHARE dataset. Similarly, we are unable to distinguish the receipt of private LTC from the receipt of publicly subsidized LTC. Moreover, our eligibility index has a binary nature, as it is built upon binary self‐reported measures of functional and cognitive limitations available in SHARE. This implies that our instrumental variable cannot predict which level of support each respondent is eligible to. Instead, it predicts whether an individual is likely to satisfy the lowest possible eligibility criteria for LTC support, based on LTC legislation.

Overall, these limitations suggest that the estimated effect of long‐term care on mental well‐being may be underestimated in our study. For example, it is plausible that older people who were already purchasing private care would see an increase in the hours of care they receive once they become eligible to public LTC. However, our model is not able to capture this increase, and would assign a null marginal effect of LTC eligibility on care use for these respondents, because they were already receiving some care without being eligible to public support. Similarly, the ability to predict eligibility levels based on an individual's medical profile would enhance the variability of the instrument, enabling a more nuanced analysis of how the intensity of public support schemes impacts well‐being.

The aforementioned data limitations also mean that we cannot identify what type of formal care is more effective in improving well‐being, for example, whether help with ADL tasks is more or less effective than other types of support. Additionally, our sample size is limited, preventing us from focusing exclusively on the oldest old, who are most likely to experience activity restrictions and higher care costs.

Second, we exploit variation across countries (and in Belgium, within two regions) in LTC eligibility rules to identify the effect of LTC on mental health. This implies, therefore, that we are not able to conduct heterogeneity analysis by country, as all respondents in a given country (or Belgian region) are subject to the same legislation. While LTC programmes are broadly similar across the countries included, some important differences remain. For example, the Belgian and Spanish systems are relatively less generous than those in France and Germany, in that they cover a lower share of population. However, the intensity of support they provide to their eligible population might still be substantial. Moreover, some programmes offer in‐kind rather than in‐cash benefits, which could also affect quality of care and therefore the well‐being outcomes for older people. Robustness tests conducted by excluding one country at a time reveal no substantial differences in the positive effects of long‐term care (LTC) on depression and other outcomes. Yet, we cannot exclude that idiosyncratic features of LTC systems, combined with epidemiological characteristics of each country population, could lead to different effects on well‐being. While our study provides an estimate on the average effect of LTC in the four countries considered, more research is needed to disentangle specific country or regional mechanisms, through larger datasets and richer information on eligibility status.

Finally, while our model includes informal care, we can only consider the care support received from children, due to the lack of instrumental variables for other care sources. While children are an important source of informal care in Europe, this approach ignores spousal care, which is often the most important source of informal care; as well as care received from relatives and friends. Further research is also needed to fully validate the exogeneity of the instrumental variables for informal care based on children characteristics. Our paper, however, is focused on the impact of formal care, and our robustness checks suggest that using different instruments for informal care does not alter our conclusions on the impact of formal care on mental health and well‐being.

Future studies should also assess the impact of LTC legislation on carers, as studies suggest that providing intensive informal care can have negative effects on the physical and mental well‐being of informal carers, and potentially lead to burnout (Bom et al. 2018; Le and Ibuka 2023; Longobardo, Rodríguez‐Sánchez, and Oliva 2023). Higher availability of formal LTC may reduce the burden of informal care, potentially also leading to wellbeing gains among informal carers (Kaschowitz and Brandt 2017; Longobardo, Rodríguez‐Sánchez, and Oliva 2023). In turn, the lack of public LTC may mean informal carers must provide more intensive care, with potentially negative consequences for their wellbeing. This might be increasingly relevant for caregivers who are facing prolonged working lives due to the rise in state pension age (Carrino, Nafilyan, and Avendano 2022), and who are employed in physically or psychosocially demanding jobs (Belloni, Carrino, and Meschi 2022). Future studies should further assess how the provision of public LTC influences the well‐being of carers, particularly adult children, and how this, in turn, may lead to intergenerational impacts on the well‐being of adult parents.

Despite these limitations, our study is one of the first to provide robust causal estimates of the net effects of publicly subsidized care on depression and quality of life in an international context. Our analysis contributes significantly to the understanding of how public care systems impact well‐being in European populations, and have important implications for policy. Depression is a leading cause of disability that has important consequences for older persons and their families, society, and health systems. And yet, there is limited evidence of effective interventions to prevent or treat depression in older age. For example, a systematic review concluded that there is no strong evidence that standard antidepressant medication, psychotherapy or combined treatments are effective for the prevention of recurrent depression in older age (Wilkinson and Izmeth 2012). Likewise, a recent review focused on adults (aged 18 years and older) with long‐term physical conditions concluded that there is limited evidence that primary care preventive interventions (psychological or pharmacological) reduce the risk of depression, particularly in the medium‐ to long‐term (Kampling et al. 2021). Our results suggest that providing home‐based care may be a cost‐effective strategy to prevent depression and improve mental health and quality of life in older people with functional limitations.

## Conflicts of Interest

The authors declare no conflicts of interest.

## Supporting information

Supporting Information S1

## Data Availability

This paper uses data from SHARE (Survey of Health, Ageing and Retirement in Europe), which are freely distributed by SHARE‐ERIC (European Research Infrastructure Consortium) to registered users through the SHARE Research Data Center (https://share‐eric.eu/data/data‐access).

## Endnotes

^1^
Our results are robust to including a full set of dummies for each ADL and iADL limitation.

^2^
Mental health conditions, such as depression, are often excluded from the assessment or assigned a very limited weight (Brugiavini et al. 2017).

^3^
Further instruments sometimes include geographic proximity of children to parents, which however is likely to represent a reverse causality relationship, as children are likely to relocate closer to older parents when a need for long term care emerges (Bergeot, Ferrari, and Gao 2024).

^4^
Our robustness tests (available upon request) also reported that using number of daughters as instrument leads to very similar results as in the main analysis. However, while this instrument has been employed in previous studies (e.g., Urwin, Lau, and Mason 2019), it partially overlaps to the information provided by the variable on number of children (because a higher number of daughters also means a higher number of children), hence making it partially exposed to the concerns expressed above.

^5^
As the Spanish LTC legislation was introduced in 2007 through a staggered implementation, we include Spanish respondents interviewed since 2007 (see Supporting Information S1: Appendix 2).

^6^
The “eligibility” status does not necessarily identify those who are actually “treated” by public programmes; furthermore, SHARE lacks information on whether or not an individual made an application for LTC benefits.

^7^
As explained in Section 4.2, the population non‐eligible to public LTC which does not receive any formal care (public or private) might be either receiving help from informal providers, or no care.

^8^
We have also employed information on the number of daughters as instrumental variable, as recently done by Urwin, Lau, and Mason (2019), and obtained very similar findings to our main specification (available upon request).

^9^
We reported estimates in real $ 2019, OECD‐PPP adjusted. The estimates for the UK include costs for depression‐specific drugs, inpatient care, other NHS services, supported accommodation, and social services; for Germany, they include inpatient and outpatient treatment costs, drugs, and nursing care (excluding informal care).
