# Supplementary material for: There Is No Place Like Home: The Impact of Public Home‐Based Care on the Mental Health and Well‐Being of Older People
Source: Health Econ. 2025 Feb 20;34(6):1085–102. doi: 10.1002/hec.4948 (PMC12045774; doi:10.1002/hec.4948)
Supplement: Supplementary file 1 — Supporting Information S1 [file HEC-34-1085-s001.docx]

# Appendix 1: measures of depression and Quality of Life

Table 1, Measures of depression (EURO-D) and Quality of Life (CASP)

| **Outcome indicator** | **SHARE survey items** | **Indicator coding** |
| --- | --- | --- |
| **CASP-total** | Aggregate of  - CASP-control  - CASP-autonomy  - CASP-pleasure  - CASP-self-realisation | Interval level, from 12 (low QOL) to 48 (high QOL) |
| **CASP-control** | - age prevents me from doing things I would like  - what happens to me is out of my control  - I feel left out of things | Interval level, from 3 (low control) to 12 (high control) |
| **CASP-autonomy** | - I can do the things I want to do  - family responsibilities prevent from doing what I want  - shortage of money prevents from doing what I want | Interval level, from 3 (low autonomy) to 12 (high autonomy) |
| **CASP-pleasure** | - I look forward to each day  - I feel that my life has meaning  - On balance I look back at life with happiness | Interval level, from 3 (low pleasure) to 12 (high pleasure) |
| **CASP-self-realisation** | - I feel full of energy  - I feel life is full of opportunities  - I feel that the future looks good for me | Interval level, from 3 (low self-realisation) to 12 (high self-realisation) |
| **EURO-D (score / caseness)** | - Sad or depressed mood in the last month  - Any hopes for the future  - Felt like would rather be dead in the last month  - Excessive guilt or self-blame  - Trouble sleeping  - Interest in things  - Irritability  - Appetite  - Fatigue  - Concentration  - Enjoyment  - Tearfulness (crying in the last month) | Interval level, from 0 (not depressed) to 12 (very depressed)  Caseness indicator for scores higher than 3 (0 = not at risk of depression, 1 = at risk of clinical depression) |

# Appendix 2: LTC programmes, eligibility rules, and matching in SHARE

*Brief summary of LTC programmes*

We provide hereafter a brief summary of the LTC programmes considered in this paper (see also [Brugiavini et al. (2017)](#_ENREF_18) and the European Commission’s Country Health Profiles for further details).

- For Belgium, we consider the in-kind programme INAMI (*National Sickness and Disability Insurance)* and the in-cash programmes APA (*Assistance to Older People*, national) and *Zorgverzekering* (for Flanders). The INAMI programme delivers in-kind services, i.e., formal home nursing care, provided irrespective of the patients’ age or income and based solely on their vulnerability conditions. The APA programme is a yearly cash-for-care benefit that ranges from roughly €1000 to €7000, which can be used by the older person to purchase care. Eligibility for APA depends on age (minimum 65 years old) and is means-tested. The *Zorgverzekering* in Flanders is a cash-for-care allowance of €135 per month that is part of a separate LTC insurance scheme. It is limited to Flemish and Brussels-Capital population, it is need-tested, yet neither age-related nor income-related.
- For France, we consider the in-kind APA (*Personalised autonomy allocation)* and the Aide Sociale. The French APA programme provides vulnerable older individuals residing in France an in-kind benefit whose intent is to finance a personalised assistance scheme, for both institutional care and home care. The level of the benefit varies according to the recipient’s health status and the level of disposable income, although means-testing plays no role in initially defining eligibility. Its monthly amount ranges roughly between €700 and € 1700. The Aide ménagère à domicile is an in-kind benefit aimed at providing home-help to older people aged 65 or older (or at least 60 years old if unable to work) who report lower degrees of dependency and are therefore not necessarily eligible for the APA allowance. The programme mainly provides support for instrumental activities of daily living.
- For Germany, we consider the in-kind/in-cash LTC insurance (Pflegeversicherung). This programme provides vulnerable individuals with benefits in cash and/or in kind, in order to ease the costs of home care assistance. Eligibility is based on need of care, regardless of their age and income. Eligible individuals can choose to receive an exclusive cash benefit, an exclusive domestic-care programme in kind, or a combination of the two. In 2017, a whole new set of eligibility rules has been implemented. The value of the monthly cash-benefit ranges between around €300 and €900, while the value of the in-kind benefit ranges between €700 and €2000.
- For Spain, we consider the the in-kind/in-cash national “System for Autonomy and Dependency Care” (SAAD), which was introduced in 2007. Eligibility to receive LTC benefits is based on the level of care a person needs in performing activities of daily living (ADLs). In-kind and in-cash benefits cannot be mixed. The system was initially introduced in 2007 to support only major dependent people, with monthly cash-benefits ranging between €400 and €500, and in-kind benefits ranging between €600 and €850. From 2011, the coverage was extended to people with a severe (i.e., less than *major*) dependency status. For this group, cash benefits range between €300 an €350, while in-kind benefits range between €400 and €430.

*Description of LTC legislation and matching with SHARE data*

We now provide details on the eligibility rules in each specific programme, and information on how we match the LTC legislations in Belgium, France, Germany and Spain to the information from the SHARE survey. We follow the methods adopted by [Brugiavini et al. (2017)](#_ENREF_6) (ch.3) and [Carrino et al. (2018)](#_ENREF_7). As stressed by [Carrino et al. (2018)](#_ENREF_7), the aim of this correspondence exercise is not to replace the work of trained assessors; rather it aims to implement legal benchmarks in a prudent and robust fashion, to identify a sub-population of “eligible individuals”.

This matching exercise faces three main limitations. First, the correspondence between the assessment-of-needs in legislations and in SHARE is not perfect: some information is not available in our data, and some medical definitions may differ slightly. Nearly all of the tasks included in the LTC legislations have a close correspondent in SHARE, and most eligibility algorithms are variously based on ADL and iADL limitations. Yet some adjustments had to be made, as will be described hereafter. Secondly, care-needs in SHARE are evaluated as binary variables (0 or 1). While this approach is consistent with the seminal [Katz et al. (1970)](#_ENREF_16) ADL and [Lawton and Brody (1969)](#_ENREF_18) taxonomies, it clashes with the approach of some LTC legislations which require more detail information on the level of intensity in the degree of activity restrictions faced by the applicant. We believe this is not a major limitation for our matching, as SHARE respondents are asked to only report difficulties that are expected to last more than three months, hence excluding low levels of activity restrictions. Lastly, the information collected in SHARE is self-reported, even though the interviewer is able to signal unreliable answers. Respondent subjectivity is, therefore, a potential issue that also affects information on the health-status, e.g., the occurrence of ADL or iADL limitations.^[[1]](#footnote-2)^

SUMMARY OF BELGIUM LTC ELIGIBILITY RULES

BELGIUM – *APA (**Aide à la Personne âgée,* *Assistance to Older People)*

The assessment process is performed through a scale (APA scale) which depicts vulnerability as determined by six items that are evaluated on a scale from 0 (no difficulties in performing the selected item) to 3 (impossibility in performing the selected item without help from others), and the overall profile of vulnerability is constructed by summing each item’ scores. We chose to assign the score of 2 whenever a respondent reports to suffer from a limitation in the corresponding SHARE task:^[[2]](#footnote-3)^

Table 2, Belgian APA and SHARE

| *Limitations* | *Value* | *SHARE tasks* |
| --- | --- | --- |
| Moving and transferring around the house | 2 out of 3 | Walking across a room *or* Getting in or out of bed |
| Preparing meals and ingesting food | 2 out of 3 | Preparing a hot meal *or* Eating (+cutting up your food) |
| Performing body-care and being able to dress | 2 out of 3 | Bathing/showering *or* Dressing (+ putting on shoes and socks) |
| Taking care of own house and performing house-tasks | 2 out of 3 | Doing work around the house *or* Managing money, such as paying bills and keeping track of expenses |
| Communication: being able to have contacts with others | 2 out of 3 | Making telephone calls |
| Need of supervision. Being able to assess and avoid dangerous situations | 2 out of 3 | Orientation in time (day, week, month, year): cannot answer three or more |

The minimum level of vulnerability corresponds to a score of 7 in the APA scale: all the applicants who get an overall index of less than 7 are not eligible to the monetary allowance. The minimum age requirement is 65 years old.

BELGIUM – *nursing home-care by INAMI/RIZIV (Institut National d’Assurance*

*Maladie-Invalidité,* *National Institute for Sickness and Disability Insurance)*

The assessment-of-need for public home help adopts a four-step scale for each item (from 1 to 4), where 0 corresponds to full-autonomy and 4 corresponds to impossibility to perform the specific task. Dependency-status on a single task arises when the need-of-care is either severe (3) or full (4). We chose to assign the score of 3 whenever a respondent reports to suffer from a limitation in the specific task.^[[3]](#footnote-4)^

Table 3, Belgian nursing home-care program and SHARE

| *Criteria* | *Value* | *SHARE tasks (binary: yes / no)* |
| --- | --- | --- |
| Washing | 3 out of 4 | Bathing or showering |
| Dressing | 3 out of 4 | Dressing (+ putting on shoes and socks) |
| Moving and transferring | 3 out of 4 | Walking across a room *or* Getting in or out of bed |
| Using the toilet | 3 out of 4 | Using the toilet (+ getting up or down) |
| Continence | 3 out of 4 | Incontinence or involuntary loss of urine |
| Eating | 3 out of 4 | Eating (+cutting up your food) |
| Orientation in time | 3 out of 4 | Orientation in time (day, week, month, year): cannot answer three or more |
| Orientation in space | 3 out of 4 | Orientation in time (day, week, month, year): cannot answer three or more |

The minimum level of vulnerability (category A) in order to be eligible corresponds to limitations in washing and dressing *or* to being disoriented in time and space (yet physically independent).

BELGIUM – Flanders supplementary LTC program *Zorgverzekering*

The programme adopts the *BEL-foto* assessment-of-need, which includes a four-step scale for each item (from 0 to 3), where 0 corresponds to full-autonomy and 3 corresponds to impossibility to perform the specific task. Since most of the health-conditions in SHARE are reported on a binary scale (yes/no) ^[[4]](#footnote-5)^, we prudently chose to assign a score of 2 in the BEL-scale to each activity that respondents report to be limited in, instead of assigning the full score of 3.

Table 4, Belgium (Flanders) Zorgverzekering and SHARE

| *Limitation* | *Value* | *SHARE tasks (binary: yes / no)* |
| --- | --- | --- |
| *Household ADL* |  |  |
| House-holding | 2 out of 3 | Doing work around the house |
| Laundry | 2 out of 3 | Doing work around the house |
| Ironing | 2 out of 3 | Doing work around the house |
| Shopping | 2 out of 3 | Shopping for groceries |
| Meal preparation | 2 out of 3 | Preparing a hot meal |
| Housework planning | 2 out of 3 | Doing work around the house |
| *Physical ADL* |  |  |
| Bathing and showering | 2 out of 3 | Bathing or showering |
| Dressing | 2 out of 3 | Dressing (+ putting on shoes and socks) |
| Functional mobility | 2 out of 3 | Getting in or out of bed |
| Using the toilet | 2 out of 3 | Using the toilet (+ getting up or down) |
| Incontinence | 2 out of 3 | Incontinence or involuntary loss of urine |
| Feeding | 2 out of 3 | Eating (+cutting up your food) |
| *Social ADL* |  |  |
| Social loss | 2 out of 3 | Reports “often” to either  How much of the time do you feel you lack companionship?; How much of the time do you feel left out?; How much of the time do you feel isolated from others? |
| Commitment to therapy and medical rules | 2 out of 3 | Taking medications |
| Safety inside/outside the house | 2 out of 3 | Doing work around the house or garden |
| Administration | 2 out of 3 | Managing money, such as paying bills and keeping track of expenses |
| Financial operations | 2 out of 3 | Managing money, such as paying bills and keeping track of expenses |
| *Mental Health* |  |  |
| Orientation in time | 2 out of 3 | Orientation in time (day, week, month, year): cannot answer three or more |
| Orientation in space | 2 out of 3 | Orientation in time (day, week, month, year): cannot answer three or more |
| Orientation in persons |  | - |
| Purposeless behavior | 2 out of 3 | - |
| Disruptive behavior | 2 out of 3 | Have you been irritable recently? |
| Lack of initiative | 2 out of 3 | Fails to mention any activity to “What have you enjoyed doing recently?” |
| Depressed mood | 2 out of 3 | In the last month, have you been sad or depressed? |
| Anxious mood | 2 out of 3 | - |

Source: Second Annex to the Ministerial Decree of 6 January 2006 regulating the determination of the severity and duration of the reduced autonomy on the basis of the BEL-profielschaal under the Flemish care insurance.

SUMMARY OF LTC ELIGIBILITY RULES IN FRANCE

FRANCE: *APA (Allocation Personnalisée d’Autonomie,* *Personalised autonomy allocation) and Aide Sociale*

Each variable (item) in the French AGGIR scale is evaluated on a three-step scale (A, B, C or 1, 2, 3), depending on the degree of limitation experienced by the patient in the specific task.^[[5]](#footnote-6)^ Since we do not have information on the intensity of the limitations reported by the SHARE respondent, we chose to prudently assign the label B (the intermediate level) whenever a respondent reports a limitation in a specific task.^[[6]](#footnote-7)^

Table 5, French AGGIR scale and SHARE

| *Discriminatory variables* | *description* | *assigned value* | *SHARE tasks* |
| --- | --- | --- | --- |
| coherence | converse or behave in a logical and sensible manner | 2 out of 3 | Orientation in time (day, week, month, year): cannot answer three or more |
| orientation | locates oneself in time and space |  |  |
| toileting | upper and lower body hygiene | 2 out of 3 | Bathing or showering |
| dressing | upper, middle and lower body dressing | 2 out of 3 | Dressing (+ putting on shoes and socks) |
| alimentation | serving and eating | 2 out of 3 | Eating (+cutting up your food) |
| evacuation | using the toilet for urine/faecal evacuations | 2 out of 3 | Using the toilet (+ getting up or down) |
| transfers | lying down, sitting down, getting up | 2 out of 3 | Getting in or out of bed |
| indoor movement | with or without technical assistance | 2 out of 3 | Walking across a room |
| outdoor movement | same as above, but outdoors | 2 out of 3 | Walking across a room *or* Using a map to figure out how to get around in a strange place |
| distant communication | using the phone and tele-alarm | 2 out of 3 | Making telephone calls |

The APA regulation requires individuals to be at least 60 years old in order to make an application for the allowance. Moreover, the AGGIR categorization of dependency must correspond to, at least, GIR 4. We went through a rather complex algorithm^[[7]](#footnote-8)^ to divide the population into 6 ISO-groups. Individuals with difficulties in - roughly - at least two ADL’s are categorized in GIR 4 regardless of their mental health status. This holds whenever the limitations are reported with at least a B (or, 2) intensity-score. It should be highlighted that being limited in “moving inside the house” is not a sufficient limitation for eligibility when the only other loss-of autonomy concerns the “transferring” task. When the “moving” limitation is selected, there should be at least one further difficulty among “using the toilet”, “dressing”, “eating” or “washing” in order to determine GIR 4. Regardless to other functional deficits in ADL, those who have cognitive impairment are assigned to, at least, GIR 2.

In order to be eligible for the Social Assistance to seniors (*Aide Sociale*), three conditions must be met by the applicant, as the next table highlights: a minimum age-requirement of 65 years old; the presence of limitations in activities related to personal hygiene and to small acts of daily livings such as meals preparation and shopping for groceries (GIR 5 or GIR 6 classification); not being a beneficiary of the Personalized Autonomy Allowance (APA) program. In order to simulate eligibility for Aide Sociale, we exploit the following SHARE information: “Bathing or showering”, “Preparing a hot meal” and “shopping for groceries”. In order to perform a prudent implementation of the regulation, we exclude the SHARE task “doing work around the house or garden”, as it seems too generic with respect to the Aide Sociale rationale.

Table 6, French Aide Sociale eligibility rules

| Aide sociale eligibility criteria | Description | SHARE information |
| --- | --- | --- |
| Age | At least 65 years old | Age |
| Vulnerability | Needing assistance with personal hygiene / meals preparation / shopping for groceries | Bathing or Showering / Preparing a hot meal / Shopping for groceries |
| Non-cumulation | Not receiving APA allowance | - |

SUMMARY OF LTC ELIGIBILITY RULES IN GERMANY

GERMANY – *Pflegeversicherung before 2017*

The assessment-of-need for the German LTC Insurance program shares some similarities with the Austrian one, in the extent to which they are both detailed and both assign to each task a measurement of need-of-care expressed in units of time. The time guidelines are not significantly different between Germany and Austria, although the former program adopts a measurement in minutes/day while the latter’s measurement unit is in hours/month. Nevertheless, the German regulation does not fix time-guidelines for the iADL limitations (which mostly correspond to the so-called “non-basic activities” in the scale): indeed, shows how some limitations have an “unspecified” time-requirement in the “need-of-care” column. The term “unspecified” refers to the fact that the need-of-care should be assessed on an individual basis by the evaluation-team. In order to be able to implement the whole German legislation on SHARE data, we chose to fill the limitations having “unspecified” requirements with the corresponding guidelines coming from the Austrian *Pflegegeld* regulation (moving inside the house, leaving and returning home, shopping, cooking, doing housework). As an example, the “cooking” task has a time-requirement of 30 hours/month (1 hour per day) in Austria, which translates into 60 minutes per day in Germany.

The minimum requirements of daily care-needs in order to be eligible (*Pflegestufe I*) are an overall need for 90’ of help, with at least 45’ attributable to basic care tasks. Following the 2012 reform, individuals affected by cognitive impairment are given access to an additional allowance, irrespective of their functional disability status (even if they are classified as *Pflegestufe 0*).^[[8]](#footnote-9)^ The SHARE survey contains information that allows us to identify cognitive-impaired individuals, with the generated variable “Orientation in time (day, week, month, year)”. In particular, cognitive deterioration is defined as not being able to answer correctly three (or more) out of the four questions.

Table 7, German Pflegeversicherung pre-2017, and SHARE

| *Basic care* | *Limitations* | *Need-of-care*  *(minutes per task)* | *Assumed daily need* | *SHARE tasks (binary: yes / no)* |
| --- | --- | --- | --- | --- |
| *✓* | Washing body (upper- lower- body, hands) | 20-25 | 40’ | Bathing or showering |
| *✓* | Dental care | 5 | 10’ | Bathing or showering |
| *✓* | Combing | 1-3 | - | Bathing or showering |
| *✓* | Shaving | 5-10 | - | Bathing or showering |
| *✓* | Taking a shower | 15-20 | 6’ | Bathing or showering |
| *✓* | Bathing | 20-25 |  |  |
| *✓* | Defecation and urination  *If also dependent for: mobility inside the house* | 8  *8+2* | 32’  *40’* | Using the toilet (+ getting up or down) |
| *✓* | Maintenance of urinary drainage bag / ostomy bag | 2-4 each | - | - |
| *✓* | Incontinence | 11 | 44’ | Incontinence or involuntary loss of urine |
| *✓* | Bite sized food preparation | 2-3 | 51’ | Eating (+cutting up your food) |
| *✓* | Food in-take | 15-20 |  |  |
| *✓* | Moving in and out of bed / changing positions | 1-3 each | 4’ | Getting in or out of bed |
| *✓* | Dressing-undressing (upper- lower body) | Unspecified | 12’ | Dressing (+ putting on shoes and socks) |
| *✓* | Moving inside house | Unspecified | (30’) | Walking across a room |
| *✓* | Standing (transferring) | Unspecified | - | Getting in or out of bed |
| *✓* | Climbing stairs | Unspecified | - | Climbing one flight of stairs without resting |
| *✓* | Leaving and returning to house | Unspecified | (20’) | Walking across a room |
| *🗶* | Shopping | Unspecified | (20’) | Shopping for groceries |
| *🗶* | Cooking | Unspecified | (60’) | Preparing a hot meal |
| *🗶* | Cleaning dwelling | Unspecified | (60’) | Doing work around the house |
| *🗶* | Washing dishes, | Unspecified |  |  |
| *🗶* | Washing and ironing clothes, | Unspecified |  |  |
| *🗶* | Managing the heating | Unspecified |  |  |
| *✓* | Cognitive limitation | Sufficient condition since 2012 | Yes | Orientation in time (day, week, month, year): cannot answer three or more |

Source: [Brugiavini et al. (2017)](#_ENREF_6). Guidelines in brackets are taken from the Austrian legislation

GERMANY – *Pflegeversicherung since 2017*

**The system post 2017**

Since 2017, following the recent reform ([Bäcker, 2016](#_ENREF_1); [BMG, 2015](#_ENREF_3); [Kalwitzki et al., 2015](#_ENREF_15)), the process of assessing individuals’ vulnerability is developed in six modules: Mobility, Cognitive abilities (mostly related to orientation, understanding and memory), Behavioural and mental problems, Limitations in ADL, Coping with illness and therapy, and Social participation. Two additional modules, “performing activities outside the house”, and “limitations in iADL” are assessed but do not contribute to the overall score nor to the eligibility decision, conversely to the pre-reform rules.

Each module includes several outcomes which are valued on a scale taking any integer value between 0 and 3, with higher numbers meaning higher dependency^^[[9]](#footnote-10)^^. Table 8 summarises the outcomes included in the assessment, together with the link we performed in the SHARE data. As usual, since in SHARE we lack information on the intensity of the limitations reported by the respondent, we assign the “most common dependency level” (e.g., 2 out of 3) when the respondent reports a limitation in a specific task.

Within the Mobility module, a close correspondence can be established. The Cognitive module involves several outcomes on orientation or understanding ability; since an outcome-specific link could be established only for some SHARE items, we chose to evaluate this module as a whole by using the “orientation” index (not being able to answer three or more questions on time – day, week, month, year) and the “recall” variable (recalling less than three words out of ten). We thus aim to reduce the effect of self-report bias and inaccuracy potentially embedded in specific respondents’ answers, and to effectively identify serious cognitive conditions through the most ‘objective’ outcomes (orientation and recall) adopted by the literature in this field ([Castro-Costa et al., 2007](#_ENREF_8)). A similar approach is followed for the Psychological section, where we adopt the EURO-D threshold of 4 points (or higher) as a more accurate predictor of latent psychological issues. The fourth module largely resembles the ADL-items covered in SHARE, except for “drinking” and “faecal incontinence”, which cannot be matched. The fifth module is primarily concerned with assessing whether the individual can cope independently with simple or elaborate illness-related requirements (e.g., taking medications, insulin injections, therapeutic activity, stoma-care routine). Each outcome is evaluated in terms of the frequency of assistance required by the applicant (daily, weekly, monthly). Since SHARE only covers the “taking medication” outcome, and no information can be retrieved on the frequency of assistance needed, this module can not be matched with the micro-data. Finally, in the module on Everyday Life we are able to match three outcomes out of six.

Table 8: German Pflegeversicherung eligibility rules since 2017

| *Limitation* | *Assigned value* | *SHARE tasks (binary: yes/no)* |
| --- | --- | --- |
| **1. Mobility** |  |  |
| Change of position in bed | 2 out of 3 | Getting in or out of bed |
| Hold the stable seating position | 2 out of 3 | Sitting for about 2 h |
| Standing up/sitting down | 2 out of 3 | Getting up from a chair after sitting for long periods |
| Move within the living area | 2 out of 3 | Walking across a room |
| Stair climbing | 2 out of 3 | Climbing one flight of stairs without resting |
| **2. Cognitive and communication** (Identify people from the surrounding area; Local orientation; Time orientation; Memory; Perform multi-step daily operations; Making decisions in everyday life; Understanding facts and information; Identify risks and hazards; Communication of elementary needs; Understanding of Prompts; Participation in a conversation) | Valued as a whole in the vulnerability scale. | Disoriented in time (day, week, month, year): cannot answer three or more Recall: less than 30% words |
| **3. Behavioural and psychological problems** (Motorised behavioural problems; Nocturnal restlessness; Self-injurious and auto aggressive behaviour; Damage to objects; Physically aggressive behaviour towards other people; Verbal aggression; Other vocal abnormalities; Defence or other supportive measures; Delusions, misunderstandings; fears; Impotence, depressive mood; Social inadequate behaviour; Other inadequate actions) | Valued as a whole in the vulnerability scale | *EURO-D score 4+* |
| **4. Dependency in ADL** |  |  |
| Wash the front upper body | 2 out of 3 | Bathing or showering |
| Combing, dental care/prosthesis cleaning, shaving | **-** | Bathing or showering |
| Wash the intimate area | 2 out of 3 | Bathing or showering |
| Showers or bathing | 2 out of 3 | Bathing or showering |
| Fitting and lining the upper & lower body | 4 out of 6 | Dressing (+ putting on shoes and socks) |
| Cutting-up the food, pouring beverages | 2 out of 3 | Eating (+ cutting up your food) |
| Eating | 6 out of 9 | Eating (+ cutting up your food) |
| Drinking | - | - |
| Use the toilet/toilet-chair | 4 out of 6 | Using the toilet (+ getting up or down) |
| Consequences of urinary incontinence, dealing with permanent catheter/urostoma | 2 out of 3 | Incontinence or involuntary loss of urine |
| Consequences of a faecal incontinence, dealing with stoma | - | - |
| **5. Dealing with illness and therapy-related requirements and stress** |  | - |
| **6. Designing everyday life and social contacts** |  |  |
| In control for planning routines and activities | - | - |
| Resting and sleeping | 2 out of 3 | Having had trouble sleeping recently |
| To keep oneself busy performing enjoyable activities | 2 out of 3 | “What have you enjoyed doing recently”—Fails to mention any enjoyable activity |
| Plan for the future (longer periods of time, make weekly or monthly schedule) | - | - |
| Interaction with people in direct contact | - | - |
| Contact management to persons outside the direct environment | 2 out of 3 | No activity performed in the last month OR Unable to use the telephone |
| Not considered for eligibility |  |  |
| **7. Out-of-home activities (iADL tasks)** |  |  |
| **8.** **Household management** (shopping for daily needs; preparation of simple meals; easy (clean) cleaning and cleaning; elaborate (heavy) clearing and cleaning; use of services; settlement of financial matters; regulation of administrative matters) |  |  |

Source: SGB XI (Buch des Sozialgesetzbuches), §14.

Within each module, the sum of outcomes is computed and then converted in an eligibility score, following guidelines and grids summarised in Table 9.

Table 9, 2017 German Pflegeversicherung correspondence grid for eligibility score

|  | Level of dependency | | | | |
| --- | --- | --- | --- | --- | --- |
|  | 0 | 1 | 2 | 3 | 4 |
|  | None | Low | Considerable | Severe | Hardest |
| Module |  |  |  |  |  |
| **1** *Mobility (sum of outcomes values)* | 0 – 1 | 2 – 3 | 4 – 5 | 6 – 9 | 10 – 15 |
| **MODULE 1 SCORE** | **0** | **2,5** | **5** | **7,5** | **10** |
| **2** Cognitive *(sum of outcomes values)* | 0 – 1 | 2 – 5 | 6 – 10 | 11 – 16 | 17 – 33 |
| *Matching with SHARE variables* |  |  |  | *Disoriented in time* | *Disoriented in time & recall less than 30%* |
| **3** Depression *(sum of outcomes values)* | 0 | 1 – 2 | 3 – 4 | 5 – 6 | 7 – 65 |
| *Matching with SHARE variables* |  |  |  | *EURO-D ≥ 4* |  |
| **MODULES 2, 3 SCORE** (the highest is considered) | **0** | **3,75** | **7,5** | **11,25** | **15** |
| **4** Dependency in ADL *(sum of outcomes values)* | 0 – 2 | 3 – 7 | 8 – 18 | 19 – 36 | 37 – 54 |
| **MODULE 4 SCORE** | **0** | **10** | **20** | **30** | **40** |
| **5** *Therapy-related requirements* | - | - | - | - | - |
| **MODULE 5 SCORE** | **0** | **5** | **10** | **15** | **20** |
| **6** *Everyday life*  *(sum of outcomes values)* | 0 | 1 – 3 | 4 – 6 | 7 – 11 | 12 – 18 |
| **MODULE 6 SCORE** | **0** | **3,75** | **7,5** | **11,256** | **15** |

Source: SGB XI (Buch des Sozialgesetzbuches), §14.

The sum of all the eligibility scores, which ranges from 0 to 100, represent the individual’s vulnerability score, which is then used to determine access to benefits. The minimum score granting access to the in-cash or in-kind benefits is 27, which corresponds to Grade of care II or *Pflegegrad 2.*

SUMMARY OF LTC ELIGIBILITY RULES IN SPAIN

SPANISH - *SAAD programme*

In 2006, the Spanish government enacted a new Law which regulates formal long-term care programmes both in the form of in-kind services and in cash benefits^^[[10]](#footnote-11)^^, to improve upon the previous system which was highly decentralised. The new programme was called “*Promoción de la Autonomía Personal y Atención a las personas en situación de dependencia*” (System for Promotion of Personal Autonomy and Assistance for Persons in a Situation of Dependency, SAAD).

The assessment-scale involves 10 *Activities* (plus one for mentally impaired individuals). Each activity comprises several *tasks*. Each Activity carries a weight (bold in Table 10, e.g., 16.8 for Eating and drinking). Each task has a coefficient (bounded between 0 and 1), representing the share of the Activity’s weight carried by that task (e.g., Cutting up food has the 20% of the Eating and drinking weight). When an individual is mentally impaired, a further eleventh Activity is considered, while the remaining ten are assigned a new weight (in parenthesis). E.g., for a mentally impaired individual the weight of the Activity Eating and drinking is 10.

Since some of the tasks included in the SAAD assessment do not have a perfect match in the SHARE dataset, we opted for the prudent approach. For example, if we consider the task “moving *outdoor”* (which is an iADL) we use from SHARE the respondents’ ability to “move *indoor”*. Since we want to avoid overestimates of the dependency status, “moving *indoor”* is a more stringent requirement than walking *outdoor* and will provide a conservative estimate of this limitation.

Table 10, Assessment of need in the Spanish SAAD

| Activities – tasks | Weight | SHARE tasks |
| --- | --- | --- |
| **Eating and drinking** | **16.8 (10)** | **Eating (+cutting up your food)** |
| Recognise e/o reach the food served | 0.25 | \| |
| Cutting up food | 0.2 | \| |
| Using cutlery | 0.3 | \| |
| Putting a glass to mouth | 0.25 | \| |
| **Control of physical needs** | **14.8 (7)** | **Using the toilet (+ getting up or down)** |
| Go to the appropriate place | 0.2 | \| |
| Dressing and undressing | 0.15 | \| |
| Adopting the right posture | 0.3 | \| |
| Cleaning oneself | 0.35 | \| |
| **Bathing** | **8.8 (8)** | **Bathing or showering** |
| Turning on and turning off taps | 0.15 | \| |
| Washing hands | 0.2 | \| |
| Using shower or bath tub | 0.15 | \| |
| Washing lower part of the body | 0.25 | \| |
| Washing upper part of the body | 0.25 | \| |
| **Other personal tasks** | **2.9 (2)** | **Bathing or showering** |
| Combing hair | 0.3 | \| |
| Cutting nails | 0.15 | \| |
| Washing hair | 0.25 | \| |
| Brushing teeth | 0.3 | \| |
| **Dressing** | **11.9 (11.6)** | **Dressing (+ putting on shoes and socks)** |
| Recognise e/o reach clothes and shoes | 0.15 | \| |
| Putting on shoes | 0.1 | \| |
| Doing up buttons | 0.15 | \| |
| Dreesing upper part of the body | 0.3 | \| |
| Dressing lower part of the body | 0.3 | \| |
| **Maintaining health** | **2.9 (11)** |  |
| Request therapeutic assistance | 0.15 | Taking medications |
| Applying therapeutic measures | 0.1 | Taking medications |
| Avoiding indoor risks | 0.25 | Walking across a room |
| Avoiding outdoor risks | 0.25 | Walking across a room |
| Distress call | 0.25 | Making telephone calls |
| **Maintaining health 2** | **9.4 (2)** | **-** |
| Changing position from lying to sitting on the bed | 0.1 | Getting in or out of bed |
| Sitting | 0.15 | Sitting for about two hours |
| Getting up from a chair | 0.1 | Getting up from a chair after sitting for long periods |
| Standing up | 0.15 | Getting in or out of bed |
| Sitting down on a chair | 0.1 | Getting in or out of bed |
| Changing posture from a sitting position | 0.1 | Getting in or out of bed |
| Changing posture from bed | 0.1 | Getting in or out of bed |
| Changing centre of gravity of body in the bed | 0.2 | Getting in or out of bed |
| **Moving inside home** | **12.3 (12.1)** | **-** |
| Movements related dressing | 0.25 | Dressing (+ putting on shoes and socks) |
| Movements related eating | 0.15 | Eating (+cutting up your food) |
| Movements related washing | 0.1 | Bathing or showering |
| Movements not related to self-care | 0.25 | Walking across a room |
| Access to all settings of the rooms | 0.1 | Walking across a room |
| Access to all rooms | 0.15 | Walking across a room |
| **Moving outside home** | **12.2 (12.9)** | **-** |
| Going out | 0.25 | Walking across a room |
| Walking around the house/buiding | 0.25 | Walking across a room |
| Walking short distances in known places | 0.2 | Walking across a room |
| Walking short distances in unknown places | 0.15 | Walking across a room *or* Using a map to figure out how to get around in a strange place |
| Walking long distances in known places | 0.1 | Walking across a room |
| Walking long distances in unknown places | 0.05 | Walking across a room *or* Using a map to figure out how to get around in a strange place |
| **Housekeeping** | **8 (8)** |  |
| Cooking | 0.45 | Preparing a hot meal |
| Shopping (for food) | 0.25 | Shopping for groceries |
| Cleaning the house | 0.2 | Doing work around the house or garden |
| Washing clothes | 0.1 | Doing work around the house or garden |
| Only for patients with a mental illness or cognitive impairment: |  |  |
| **Making decisions** | **(15.4)** | **Disoriented in time (day, week, month, year): cannot answer three or more** |
| Source: Real Decreto 174/2011, Ministerio de Sanidad, Politica Social e Igualdad "BOE", num.42, 18/02/2011 | | |

The final score is the sum of the weights of the tasks for which the individual has difficulty, multiplied by the degree of supervision required and the weight assigned to that activity:

Score = ∑ (Weight of the task performed with difficulty * Degree of supervision required in the specific task * Weight of the corresponding activity)

The Spanish legislation allows for different degrees of supervision required, which depend on the intensity of the *loss-of-autonomy* experienced in each task (). The need can be *special*, *full* or *partial*, corresponding to coefficients of 1, 0.95 or 0.9 respectively. We prudently selected a need-of-support equal to 0.9.

Three levels of eligibility are identified, depending on the need-score, and each level is split in two parts, representing lower and higher levels of need (): (i) *moderate dependency 1 and 2*; (ii) *severe dependency 1 and 2*; and (iii) *major dependency 1 and 2* ([Fernanda Gutierrez et al., 2010](#_ENREF_13); [Jiménez-Martín and Prieto, 2010](#_ENREF_14)). The reform was implemented in steps, also due to the Great Recession, and individuals in the highest levels of vulnerability were given priority. Before 2011, eligibility was only assigned for *Major dependency 1 and 2*; from 2011, eligibility also included *Severe dependency 1 and 2* ([Costa-Font et al., 2018](#_ENREF_10)). Although the new system was supposed to be fully operational by 2015, delays were imposed by the austerity policies which generated a “dependency limbo” category of applicants, i.e., individuals who were assessed as eligible and yet not receiving any benefit *(*[Peña-Longobardo et al., 2016](#_ENREF_22)*).* Hence, we prudently decided to follow [Costa-Font et al. (2018)](#_ENREF_10) and assign the eligibility as follows. Before 2011 (which in our analysis corresponds to SHARE wave 2, as we do not use wave 3 or wave 4, and as the Spanish rules were not in place during wave 1) we assign the eligibility to *Major dependence 1 and 2*. For the years after 2011, which in our analysis correspond to Wave 5 and subsequent waves, we assign the eligibility for *Major* and *Severe dependence (1 and 2)*.

Table 11, Degrees and Levels of Dependency (score) in the Spanish SAAD

| Degree | Score | Available from |
| --- | --- | --- |
| Major dependence -2 | 90-100 | 2007 (SHARE Wave 2) |
| Major dependence -1 | 75-89 | 2007 (SHARE Wave 2) |
| Severe dependence -2 | 65-74 | 2013 (SHARE Wave 5) |
| Severe dependence -1 | 50-64 | 2013 (SHARE Wave 5) |
| Moderate dependence -2 | 40-49 |  |
| Moderate dependence -1 | 25-39 |  |
| Not dependent | 0-24 |  |

Source: Gutierrez et al., 2010.

# Appendix 3: Sensitivity tests

*SES heterogeneity*

We tested whether the impact of formal care on mental health differs depending on the recipient’s socioeconomic status (SES). This is important for two reasons. First, the importance of different mechanisms through which long-term care impacts mental health may differ by SES. For example, the effect of home-based care provision on loneliness might be larger for low-SES than for high-SES groups, given that persons with a lower SES have smaller social networks in later life ([Nazroo, 2017](#_ENREF_20)), and the marginal effect of care is larger for adults with lower levels of social integration ([Silverstein et al., 1996](#_ENREF_24)). Second, our instrument aims to capture an exogenous increase in the use of long-term care, as being eligible to public programmes of LTC is a required condition to receive publicly subsidised home-based care. Given larger budget constrains for individuals with a lower SES, we expect the impact of becoming eligible to subsidised home-based care to have a larger impact in socioeconomically constrained households, as they are less likely to purchase care on the market than households with a higher SES.

We estimate IV models for different SES groups defined by (i) household wealth and (ii) higher than median household income. As shown in Figure 1, the overall effect of care on mental health is positive and similar for all SES groups. Similar results (not shown) are obtained for education (respondents with upper-secondary education or less, versus respondents with more than upper-secondary education). However, further research employing a more detailed variable on care use (e.g., hours of care) might allow to disentangle SES differences, e.g., for different levels of care intensity.

Figure 1, effect of formal home-based care use on EURO-D depression score: heterogeneity by SES

Note: The figure illustrates the coefficients estimated for “any care use” from model (1), separately for high- and low-SES groups, defined with respect to country-specific median income and wealth in the sample. Sample: individuals aged 60+, with 1+children, in SHARE waves 1,2,5-7 in Belgium, France, Germany and Spain. Standard errors are clustered by NUTS-1 regions (57). Full results available from the authors.

Table 12, effect of receiving formal home care on depression score (instrumental variable model)

| Dependent variable: EURO-D score | SES Sub-group | | | |
| --- | --- | --- | --- | --- |
|  | Low wealth | High wealth | Low income | High income |
|  |  |  |  |  |
| Any formal home care | -2.924*** | -2.137 | -2.924*** | -2.137 |
|  | (0.824) | (1.349) | (0.824) | (1.349) |
|  |  |  |  |  |
| N | 18,570 | 14,608 | 18,570 | 14,608 |

Note: we report the marginal effect coefficients for “any formal care use” estimated using model (1) with instrumental variables. Sample: individuals aged 60+, with 1+children, in SHARE waves 1,2,5-7 in Belgium, France, Germany and Spain. Controls: informal care use (instrumented), age, age (quadratic), gender, living arrangements, education, living area, self-reported health, ADL limitations, iADL limitations, mobility limitations, ADL limitation * mobility limitations, cognitive health, fixed effects for household income (quintiles), waves and NUTS-1 regions. Standard errors are clustered by NUTS-1 regions (57). Statistical significance: * p<0.1, ** p<0.05, *** p<0.01 .

*LTC eligibility and placebo outcomes*

We first tested, through model (2), whether our LTC eligibility indicator has predictive power towards outcomes which we would not expect to be influenced by eligibility. As shown in Table 13, we find no statistically significant effect of LTC eligibility on sociodemographic characteristics such as number of children, amount of household income and wealth, low education, probability of living in rural areas, and probability of living in a couple. Although the exogeneity of our instrument is an assumption that cannot be directly tested, these results support the hypothesis that, after controlling for health and functional measures, our index is uncorrelated with personal characteristics potentially associated with the decision to use care.

Table 13, ‘placebo’ effect of LTC eligibility on personal characteristics

|  | (1)  n. of children | (2)  Log household income | (3)  Log household wealth | (4)  Being in low education | (5)  Living in rural area | (6)  Living in couple |
| --- | --- | --- | --- | --- | --- | --- |
|  |  |  |  |  |  |  |
| Eligible for public LTC | 0.007 | -0.022 | 0.051 | -0.014 | -0.016 | -0.016 |
|  | (0.059) | (0.031) | (0.194) | (0.027) | (0.016) | (0.016) |
|  |  |  |  |  |  |  |
| N | 33,178 | 33,178 | 29,827 | 33,178 | 33,178 | 33,178 |
|  |  |  |  |  |  |  |

Note: results from model (2). Sample: individuals aged 65+, with 1+children, in SHARE waves 1,2,5-7 in Belgium, France, Germany and Spain. Controls: self-reported health, ADL limitations, iADL limitations, mobility limitations, cognitive health, fixed effects for waves and NUTS-1 regions. Standard errors are clustered by regions (57). Statistical significance: * p<0.1, ** p<0.05, *** p<0.01 . Full results available from the authors.

*Alternative models for health variables and sample selections*

We estimated the sensitivity of our findings to different specifications of the empirical model, and different sample selections.

In our main specification, we include ADL and iADL as count variables. However, specific functional limitations may differently affect individuals' care use and well-being, and are weighted differently in some LTC legislations ([Carrino et al., 2018](#_ENREF_7)). Hence, we re-estimated our model including a full set of dummies for each ADL and iADL limitation. Our results were nearly identical to those in our main specification.

It is possible that our results are driven by the oldest old in our sample, who typically have the highest level of needs and the highest rates of long-term care utilisation. To assess this, we re-estimate our models in a sample that excludes respondents aged 90 and over. Our results for depressive symptoms, quality of life and loneliness are very similar to those for the full sample. Our results suggest that the beneficial effect of LTC is larger for people below the age of 90. This is not surprising as our instrumental variable, which provides exogenous variation to formal care use, is eligibility to receive any care-support from the government, including the lowest and basic categories of eligibility. We believe that, for these reasons, the instrument is well suited to capture the effect of receiving support in a relatively early stage of functional decline, rather than in a more severe stage.

Table 14, Sensitivity checks on health measures and oldest old sample

|  | (i)  EURO-D | | (ii)  EURO-D caseness | | (iii)  CASP caseness | | (iv)  Loneliness caseness | | |
| --- | --- | --- | --- | --- | --- | --- | --- | --- | --- |
| Add single ADL & iADL |  |  |  |  |  |  |  |  |  |
| Formal care | -4.192*** | (1.059) | -0.144*** | (0.026) | 0.107*** | (0.036) | -0.062*** | (0.014) |  |
| Informal care | -0.534 | (1.359) | -0.096*** | (0.032) | 0.176*** | (0.033) | -0.063*** | (0.022) |  |
| N | 33,178 |  | 33,178 |  | 29,703 |  | 22,346 |  |  |
|  |  |  |  |  |  |  |  |  |  |
| Exclude self-rated health |  |  |  |  |  |  |  |  |  |
| Formal care | -2.31** | (1.061) | -0.154*** | (0.018) | 0.174*** | (0.036) | -0.075*** | (0.012) |  |
| Informal care | 0.695 | (1.433) | -0.096*** | (0.025) | 0.176*** | (0.034) | -0.069*** | (0.023) |  |
| N | 33,178 |  | 33,178 |  | 29,703 |  | 23,246 |  |  |
|  |  |  |  |  |  |  |  |  |  |
| Excluding aged 90+ |  |  |  |  |  |  |  |  |  |
| Formal care | -3.111*** | (1.079) | -0.149*** | (0.019) | 0.168*** | (0.035) | -0.063*** | (0.015) |  |
| Informal care | 0.610 | (1.479) | -0.077** | (0.024) | 0.145*** | (0.037) | -0.062** | (0.024) |  |
| N | 32,643 |  | 32,643 |  | 29,224 |  | 22,871 |  |  |
|  |  |  |  |  |  |  |  |  |  |
|  |  |  |  |  |  |  |  |  |  |

Note: we report the marginal effect coefficients for “any formal care use” and “any informal care use” estimated using model (1) with instrumental variables, and with the sensitivity check described in the first column of the table. Sample: individuals aged 60+, with 1+children, in SHARE waves 1,2,5-7 in Belgium, France, Germany and Spain. Controls: age (quadratic), gender, living arrangements, education, living area, self-reported health, ADL limitations, iADL limitations, mobility limitations, cognitive health, fixed effects for household income and wealth (quintiles), waves and NUTS-1 regions. Standard errors are clustered by NUTS-1 regions (57). Statistical significance: * p<0.1, ** p<0.05, *** p<0.01 . Full results available from the authors.

We further test the sensitivity of our findings to alternative sample selections based on the age of respondents. As explained in the main text, our main sample selection includes respondents aged 60 or older. Many studies, however, focus on a slightly older population, considering respondents aged 65 or older. Our results are confirmed when restricting the sample to respondents aged 65 or older, as summarized in Table 15. This selection would exclude eligible respondents in France, Germany and Spain (and partially in Belgium), who can claim a LTC benefit when aged younger than 65. The proportion of respondents who are deemed eligible to receive LTC benefits is 2.8%, while around 2.7% report receiving formal care at home. These proportions are not negligible when compared to the proportions of eligible (8.5%) and formal care users (8.6%) among the 65+ population.

Table 15, Sensitivity test: sample restricted to respondents aged 65+

| IV Estimates | (1)  EURO-D score | (2)  EURO-D caseness | (3)  CASP  score | (4)  CASP Control | (5)  CASP Autonomy | (6)  CASP  Self-realisation | (7)  CASP pleasure | (8)  CASP median caseness | (9)  Loneliness caseness |
| --- | --- | --- | --- | --- | --- | --- | --- | --- | --- |
|  |  |  |  |  |  |  |  |  |  |
| Any formal home care | -2.164** | -0.129*** | 1.265 | 1.665** | 0.352 | -0.628 | 0.265 | 0.152*** | -0.063*** |
|  | (0.915) | (0.024) | (2.127) | (0.813) | (0.851) | (0.745) | (0.707) | (0.033) | (0.019) |
| Any informal care (children) | -1.145 | -0.062** | 0.464 | 0.705 | -4.391*** | 1.902 | 2.687** | 0.142*** | -0.066** |
|  | (1.308) | (0.031) | (3.473) | (1.284) | (1.594) | (1.353) | (1.088) | (0.041) | (0.026) |
|  |  |  |  |  |  |  |  |  |  |
| SW F-test  FC equation | 15.6 | 15.6 | 17.5 | 17.5 | 17.5 | 17.5 | 17.5 | 17.5 | 23.7 |
| SW F-test  IC equation | 28.7 | 28.7 | 16.3 | 16.3 | 16.3 | 16.3 | 16.3 | 16.3 | 10.4 |
| N | 24,857 | 24,857 | 22,273 | 22,273 | 22,273 | 22,273 | 22,273 | 22,273 | 16,755 |

Note: we report the marginal effect coefficients for “any formal care use” and “any informal care use” estimated using model (1) with instrumental variables. Sample: individuals aged 65+, with 1+children, in SHARE waves 1,2,5-7 in Belgium, France, Germany and Spain. Controls: age (quadratic), gender, living arrangements, education, living area, self-reported health, ADL limitations, iADL limitations, mobility limitations, cognitive health, fixed effects for household income and wealth (quintiles), waves and NUTS-1 regions. Standard errors are clustered by NUTS-1 regions (57). Full results available from the authors. Statistical significance: * p<0.1, ** p<0.05, *** p<0.01 .

As the prevalence of loss of autonomy tends to increase with age, it would be important to understand whether the impact of formal care on health and well-being is significantly different between the oldest old and relatively younger people eligible to LTC support. Answering this research question is not feasible in our dataset, as it would require a larger sample size than currently available. We nevertheless attempted to estimate our model for a subsample of respondents aged 70 or older. Although the sample size becomes small and some estimates become less precise, the beneficial effect of Long-Term Care use on depression and quality of life is confirmed, as summarized in Table 16. The estimated coefficients are not statistically different than those obtained on the whole sample. Due to the reduced sample size, it has not been possible to estimate the model on loneliness.

Table 16: Sensitivity test: sample restricted to respondents aged 70+

| IV Estimates | (1)  EURO-D score | (2)  EURO-D caseness | (3)  CASP  score | (4)  CASP Control | (5)  CASP Autonomy | (6)  CASP  Self-realisation | (7)  CASP pleasure | (8)  CASP median caseness |
| --- | --- | --- | --- | --- | --- | --- | --- | --- |
|  |  |  |  |  |  |  |  |  |
| Any formal home care | -1.849* | -0.145*** | -0.498 | 1.327* | -0.254 | -1.063 | 0.248 | 0.162*** |
|  | (1.070) | (0.036) | (2.591) | (0.821) | (0.689) | (1.009) | (0.791) | (0.055) |
| Any informal care (children) | -1.724 | -0.036 | 6.391* | 1.677 | -2.545* | 2.563* | 2.651** | 0.104 |
|  | (1.321) | (0.047) | (3.318) | (1.275) | (1.451) | (1.539) | (1.153) | (0.074) |
|  |  |  |  |  |  |  |  |  |
| SW F-test instruments for FC equation | 10.34 | 10.34 | 12.40 | 12.40 | 12.40 | 12.40 | 12.40 | 12.40 |
| SW F-test instruments for IC equation | 27.97 | 27.97 | 18.30 | 18.30 | 18.30 | 18.30 | 18.30 | 18.30 |
| N | 16,485 | 16,485 | 13,592 | 13,592 | 13,592 | 13,592 | 13,592 | 13,592 |
| Sample Average |  |  |  |  |  |  |  |  |

Note: we report the marginal effect coefficients for “any formal care use” and “any informal care use” estimated using model (1) with instrumental variables. Sample: individuals aged 70+, with 1+children, in SHARE waves 1,2,5-7 in Belgium, France, Germany and Spain. Controls: age (quadratic), gender, living arrangements, education, living area, self-reported health, ADL limitations, iADL limitations, mobility limitations, cognitive health, fixed effects for household income and wealth (quintiles), waves and NUTS-1 regions. Standard errors are clustered by NUTS-1 regions (57). Full results available from the authors. Statistical significance: * p<0.1, ** p<0.05, *** p<0.01 .

*Alternative instrumental variable for informal care use*

As discussed in the main paper, we implement alternative strategies to ensure that our findings on the effect of formal care use on health and well-being are not driven by the choice of the instrumental variable for informal care use. First, we re-estimate our model using only information on the proportion of daughters as the single instrument for informal care, as in [Bonsang (2009)](#_ENREF_4). We exclude the variable on number of children to address concerns that it might affect the parent’s psychological wellbeing through other channels than informal care support.

Table 17 reports the results for our full model when informal care is instrumented solely with information on the share of daughters a respondent has.

Table 17, effect of care use on EURO-D score, when instrumenting informal care with fraction of daughters

|  | (1)  any formal home care | (2)  any informal care (from children) | (3)  EURO-D  OLS | (4)  EURO-D  IV |
| --- | --- | --- | --- | --- |
| Eligible for home care | 0.120*** | 0.016 | - | - |
|  | (0.001) | (0.015) |  |  |
| Fraction of daughters | 0.001 | 0.0015*** | - | - |
|  | (0.001) | (0.0001) |  |  |
|  |  |  |  |  |
| Any formal home care | - | - | 0.154*** | -2.391*** |
|  |  |  | (0.042) | (1.016) |
| Any informal care (children) | - | - | 0.130** | -2.060 |
|  |  |  | (0.049) | (3.127) |
| age | -0.035*** | -0.034*** | 0.123*** | -0.150* |
|  | (0.005) | (0.005) | (0.045) | (0.087) |
| age^2^ | 0.002*** | 0.001*** | -0.001*** | 0.001* |
|  | (0.000) | (0.000) | (0.000) | (0.001) |
| female | -0.001 | 0.020*** | 0.635*** | 0.661*** |
|  | (0.003) | (0.003) | (0.050) | (0.062) |
| in couple | -0.030*** | -0.089*** | -0.153*** | -0.472** |
|  | (0.004) | (0.007) | (0.034) | (0.234) |
| low educ (ref.:high) | 0.006 | 0.008 | 0.287*** | 0.290*** |
|  | (0.005) | (0.005) | (0.045) | (0.048) |
| mid educ (ref: high) | 0.003 | 0.005 | 0.061* | 0.063 |
|  | (0.004) | (0.006) | (0.036) | (0.040) |
| bad subject. health | 0.025*** | 0.027*** | 1.437*** | 1.736*** |
|  | (0.007) | (0.009) | (0.058) | (0.100) |
| # adl | 0.049** | 0.039** | 0.292*** | 0.601** |
|  | (0.027) | (0.019) | (0.101) | (0.245) |
| # iadl | 0.045*** | 0.053*** | 0.236*** | 0.534*** |
|  | (0.006) | (0.005) | (0.021) | (0.133) |
| any mobility limit. | 0.009*** | 0.092*** | 0.672*** | 1.092*** |
|  | (0.003) | (0.006) | (0.037) | (0.231) |
| low cognitive health | -0.013 | 0.006 | 0.422*** | 0.568*** |
|  | (0.009) | (0.009) | (0.061) | (0.070) |
| #adl * mobility limit. | -0.020 | -0.040** | -0.165 | -0.345 |
|  | (0.026) | (0.019) | (0.130) | (0.244) |
|  |  |  |  |  |
| *F-test -* Eligible for home care | 36.8 | 1.2 |  |  |
| *F-test -* Fraction of daughters | 2.7 | 10.4 |  |  |
| Sanderson-Windmeijer F-test of excluded instruments | 24.1*** | 11.6*** |  |  |
| N | 33,178 | 33,178 | 33,178 | 33,178 |
| Sample Average | 0.076 | 0.101 | 2.508 | 2.508 |

Note: we report the marginal effect coefficients for “any formal care use” and “any informal care use” estimated using model (1) with instrumental variables, where informal care is instrumented with fraction of daughters. Sample: individuals aged 60+, with 1+children, in SHARE waves 1,2,5-7 in Belgium, France, Germany and Spain. Controls: age (quadratic), gender, living arrangements, education, living area, self-reported health, ADL limitations, iADL limitations, mobility limitations, cognitive health, fixed effects for household income and wealth (quintiles), waves and NUTS-1 regions. Standard errors are clustered by NUTS-1 regions (57). Full results available from the authors. Statistical significance: * p<0.1, ** p<0.05, *** p<0.01 .

Overall, this approach lowers the predictive power of the instrument for informal care, with respect to using number of children among the instrumental variables, but it reduces the risk of confounding factors related to the number of children, such as income or loneliness.

As shown in Table 17, formal care is estimated to have a meaningful beneficial effect on EURO-D score, which is close to what we obtained in the full model which included number of children as instrument for informal care. Similarly, as reported in Table 18, formal care is estimated to reduce the risk of clinical depression by 13.8 percentage points (column 1), while increasing the probability of reporting a higher than median quality of life score (column 7) by 14.8 percentage points. As in our main findings, we report an improvement in the feelings of having control over one’s own life (column 4), and a reduction in the risk of reporting feeling of loneliness (column 8). We also note that receiving informal care from children is linked to lower risk of depression as well as higher probability of reporting higher-than-median Quality of Life, and lower probability of reporting feelings of loneliness.

Table 18, robustness test using fraction of daughters as instrumental variable. Outcomes: risk of depression, Quality of Life, and risk of loneliness.

| IV Estimates | (1)  EURO-D caseness | (2)  CASP  score | (3)  CASP Control | (4)  CASP Autonomy | (5)  CASP  Self-realisation | (6)  CASP pleasure | (7)  CASP median caseness | (8)  Loneliness caseness |
| --- | --- | --- | --- | --- | --- | --- | --- | --- |
|  |  |  |  |  |  |  |  |  |
| Any formal home care | -0.138*** | 1.433 | 1.637** | 0.934 | -0.550 | 0.199 | 0.148*** | -0.067*** |
|  | (0.020) | (2.172) | (0.775) | (0.830) | (1.129) | (0.665) | (0.034) | (0.013) |
| Any informal care (children) | -0.078** | 4.004 | -0.185 | -4.361 | 4.572 | 1.036 | 0.159*** | -0.066** |
|  | (0.035) | (11.52) | (2.922) | (3.571) | (3.915) | (0.992) | (0.035) | (0.025) |
|  |  |  |  |  |  |  |  |  |
| SW F-test instruments for FC equation | 24.1 | 23 | 23 | 23 | 23 | 23 | 23 | 38.1 |
| SW F-test instruments for IC equation | 11.6 | 6.8 | 6.8 | 6.8 | 6.8 | 6.8 | 6.8 | 4 |
| N | 33,178 | 29,703 | 29,703 | 29,703 | 29,703 | 29,703 | 29,703 | 23,246 |
| Sample Average | 0.282 | 37.7 | 8.67 | 9.26 | 9.38 | 10.45 | 0.5 | 0.112 |

Note: we report the marginal effect coefficients for “any formal care use” and “any informal care use” estimated using model (1) with instrumental variables, where informal care is instrumented with fraction of daughters. Sample: individuals aged 60+, with 1+children, in SHARE waves 1,2,5-7 in Belgium, France, Germany and Spain. Controls: age (quadratic), gender, living arrangements, education, living area, self-reported health, ADL limitations, iADL limitations, mobility limitations, cognitive health, fixed effects for household income and wealth (quintiles), waves and NUTS-1 regions. Standard errors are clustered by NUTS-1 regions (57). Statistical significance: * p<0.1, ** p<0.05, *** p<0.01 . Full results available from the authors.

Finally, we have re-estimated the robustness tests as in Table 14, Table 15 and Table 16, while including only fraction of daughters as instrumental variable for informal care, and we obtained very similar results that confirm the beneficial impact of publicly subsidised formal care use on psychological health and well-being (available upon request).

Second, as a further robustness test, we exclude information on informal care from the set of regressors, while including children characteristics such as proportion of daughters and number of children. As summarized in Table 19, all results confirm the beneficial effect of formal care use on risk of depression, quality of life, and risk of loneliness.

Table 19, effect of formal care on outcomes, excluding informal care from the set of regressors.

| Model without informal care variable (controlling for children characteristics) | (i)  EURO-D | | (ii)  EURO-D caseness | | (iii)  CASP caseness | | (iv)  Loneliness caseness | |  |
| --- | --- | --- | --- | --- | --- | --- | --- | --- | --- |
|  |  |  |  |  |  |  |  |  | |
| Formal care | -3.001*** | (0.675) | -0.166*** | (0.017) | 0.199*** | (0.031) | -0.076*** | (0.014) | |
| N | 33,178 |  | 33,178 |  | 29,703 |  | 22,346 |  | |
|  |  |  |  |  |  |  |  |  | |

Note: we report the marginal effect coefficients for “any formal care use” estimated using model (1) but excluding informal care from the set of regressors, and replacing it with variables on children characteristics (number of children, fraction of daughters). Formal care is instrumented with the eligibility index, as in previous models. Sample: individuals aged 60+, with 1+children, in SHARE waves 1,2,5-7 in Belgium, France, Germany and Spain. Controls: age (quadratic), gender, living arrangements, education, living area, self-reported health, ADL limitations, iADL limitations, mobility limitations, cognitive health, fixed effects for household income and wealth (quintiles), waves and NUTS-1 regions. Standard errors are clustered by NUTS-1 regions (57). Statistical significance: * p<0.1, ** p<0.05, *** p<0.01 . Full results available from the authors.

*Country heterogeneity*

Our sample size does not allow us to study the heterogeneity of the effect of formal LTC use on health and well-being in different country contexts, especially given the relative low occurrence of formal care use and eligibility entitlement in the population. As explained in the methods, a source of causal identification for our study relies in the comparison of otherwise similar individuals who are subject to different eligibility rules because they live in different areas; while another source of identification relies on the non-linear nature of eligibility algorithms that grant different eligibility status to similarly sick individuals who have different combination of functional and cognitive difficulties. When focusing on country-specific data, the international source of causal identification is removed, and richer data are needed to support the identification strategy, for example, through studying regional-level data on care use, combined with an eligibility index that either varies over time / across regions (within a country), or that at least includes information on levels of eligibility on an intensity scale.

We conducted a tentative test of country heterogeneity, by re-estimating our models after excluding one country at a time from the sample. Figure 2 summarizes the estimates obtained for the effect of formal care on depression, quality of life and loneliness outcomes (full results available upon request). Overall, the beneficial effects of receiving formal care on depression scores, risk of depression, quality of life and loneliness are confirmed and are found not be statistically different across specifications. The availability of richer data might allow to study whether, for example, the beneficial effect on Quality of Life and risk of loneliness is more pronounced in Belgium than elsewhere.

Figure 2, effect of formal care use on outcomes, when excluding one country at a time from the sample

**

**

Note: we plot the marginal effect coefficients for “any formal care use” on health outcomes, estimated using model (1) with instrumental variables. Sample: individuals aged 60+, with 1+children, in SHARE waves 1,2,5-7 in Belgium, France, Germany and Spain. Controls: age (quadratic), gender, living arrangements, education, living area, self-reported health, ADL limitations, iADL limitations, mobility limitations, cognitive health, fixed effects for household income and wealth (quintiles), waves and NUTS-1 regions. Standard errors are clustered by NUTS-1 regions (57). Full results available from the authors.

# Appendix 4: Further mechanisms analysis

We further explored potential mechanisms through which access to publicly subsidised formal home-care can improve mental health and quality of life.

Several studies have documented that formal care use can reduce the risk of hospitalisation. First, to the extent to which access to subsidised LTC support reduces risk of loneliness, it is predicted to reduce the risk of hospitalisation ([Molloy et al., 2010](#_ENREF_19); [Rapp et al., 2015](#_ENREF_23)). Second, access to publicly subsided support has been shown to increase care quality (better disease management, higher investment in formal non-medical care), which can in turn lead to better mental health and lower risk of hospitalisation. For example, access to LTC support can lead to the implementations of home adjustments or alterations which can improve quality of life ([Costa-Font et al., 2018](#_ENREF_10)).

Moreover, to the extent to which enhanced access to publicly subsidised formal LTC programmes is able to reduce caregiver burden, it can help improving the quality of informal care, which in turn could lead to better mental health and lower hospitalisation ([Costa-Font et al., 2018](#_ENREF_10); [Rapp et al., 2015](#_ENREF_23)).

We tested the link between formal care use and hospitalisation, exploiting a specific question in the SHARE survey which asks respondents whether, in the previous twelve months, they have been in a hospital overnight. The overall prevalence of respondents reporting an overnight hospital stay in our sample is 17.3%.

In Table 20, we show that, in the instrumental variable model, formal care use is linked to a reduction in the risk of hospitalisation by 4.3 percentage points (column i), but this effect is not statistically significant. We tried to estimate an alternative model (column ii), as done in a previous robustness test whose results are shown in Table 14, which includes a full set of indicators for ADL and iADL limitations. In this model, the effect of receiving formal care on risk of hospitalisation is more precisely estimated at a reduction of 5.2 percentage points, which is statistically significant at 10 percent level. These results should therefore be interpreted with caution.

We also assessed whether access to publicly subsidised LTC increases the probability of reporting living in a house with “special features that assist persons who have physical impairments or health problems”. We find that, in the instrumental variable model (column iii), receiving publicly subsidised formal care increases reporting living in a house with home adaptations by 10 percentage points (p=0.053). This result supports the theory that access to more formally provided LTC may enhance quality of life through improved housing conditions.

Our data are limited to older individuals receiving care, and we lack information on the mental well-being or burnout of their adult children. Therefore, we cannot test a direct mechanism between access to formal care and reduction in caregivers’ burden.

Table 20, Sensitivity checks on risk of hospitalization and home adaptation

|  | (i)  Risk of hospitalisation | | (ii)  Risk of hospitalisation (with full set of ADL and iADL dummies) | | (iii)  Living in house with special features | |
| --- | --- | --- | --- | --- | --- | --- |
|  |  |  |  |  |  |  |
| Formal care | -0.043 | (0.028) | -0.052* | (0.028) | 0.101* | (0.052) |
| Informal care | -0.044 | (0.024) | -0.043* | (0.024) | 0.046 | (0.041) |
|  |  |  |  |  |  |  |
| N | 33,554 |  | 33,554 |  | 32,781 |  |
|  |  |  |  |  |  |  |
| Sample average | 0.173 |  | 0.173 |  | 0.142 |  |
|  |  |  |  |  |  |  |
|  |  |  |  |  |  |  |

Note: we report the marginal effect coefficients for “any formal care use” and “any informal care use” estimated using model (1) with instrumental variables, and with the sensitivity check described in the first column of the table. Sample: individuals aged 60+, with 1+children, in SHARE waves 1,2,5-7 in Belgium, France, Germany and Spain. Controls: age (quadratic), gender, living arrangements, education, living area, self-reported health, ADL limitations, iADL limitations, mobility limitations, cognitive health, fixed effects for household income and wealth (quintiles), waves and NUTS-1 regions. In column (ii), we include a full set of indicators for ADL and iADL limitations. Standard errors are clustered by NUTS-1 regions (57). Statistical significance: * p<0.1, ** p<0.05, *** p<0.01 . Full results available from the authors.

REFERENCES

Bäcker, G. (2016). 'Reform of the long-term care insurance in Germany', *ESPN Flash Report*, vol. **2016/43**.

Baker, M., M. Stabile and C. Deri (2004). 'What do self-reported, objective, measures of health measure?', *Journal of Human Resources*, vol. **39**(**4**), pp. 1067-1093.

BMG (2015). 'Gesetzentwurf der Bundesregierung. Entwurf eines Zweiten Gesetzes zur Stärkung der pflegerischen Versorgung und zur Änderung weiterer Vorschriften (Zweites Pflegestärkungsgesetz – PSG II).', *Bundesministerium für Gesundheit*.

Bonsang, E. (2009). 'Does informal care from children to their elderly parents substitute for formal care in Europe?', *Journal of health economics*, vol. **28**(**1**), pp. 143-154.

Bound, J. (1991). 'Self-Reported Versus Objective Measures of Health in Retirement Models', *Journal of Human Resources*, pp. 106-138.

Brugiavini, A., L. Carrino, C. E. Orso and G. Pasini (2017). *Vulnerability and Long-term Care in Europe: an Economic perspective* London: Palgrave MacMillan.

Carrino, L., C. E. Orso and G. Pasini (2018). 'Demand of long‐term care and benefit eligibility across European countries', *Health economics*.

Castro-Costa, E., M. Dewey, R. Stewart, S. Banerjee, F. Huppert, C. Mendonca-Lima, C. Bula, F. Reisches, J. Wancata, K. Ritchie, M. Tsolaki, R. Mateos and M. Prince (2007). 'Prevalence of depressive symptoms and syndromes in later life in ten European countries: the SHARE study', *Br J Psychiatry*, vol. **191**, pp. 393-401.

Chan, K. S., J. D. Kasper, J. Brandt and L. E. Pezzin (2012). 'Measurement Equivalence in ADL and IADL Difficulty Across International Surveys of Aging: Findings From the HRS, SHARE, and ELSA', *The Journals of Gerontology Series B: Psychological Sciences and Social Sciences*, vol. **67B**(**1**), pp. 121-132.

Costa-Font, J., S. Jimenez-Martin and C. Vilaplana (2018). 'Does long-term care subsidization reduce hospital admissions and utilization?', *Journal of health economics*, vol. **58**, pp. 43-66.

Dupourqué, E., S. Schoonveld and J. B. Bushey (2012). 'AGGIR, the Work of Grids', *Long-term Care News*, vol. **32**.

Dwyer, D. S. and O. S. Mitchell (1999). 'Health problems as determinants of retirement: Are self-rated measures endogenous?', *Journal of health economics*, vol. **18**(**2**), pp. 173-193.

Fernanda Gutierrez, M., S. Jimenez-Martin, R. Vegas Sanchez and C. Vilaplana (2010). 'The Spanish Long-term Care System. ENEPRI Research Report No. 88, 15 June 2010'.

Jiménez-Martín, S. and C. V. Prieto (2010). 'A double sample selection model for unmet needs, formal care and informal caregiving hours of dependent people in Spain', *Documento de Trabajo*, vol. **2010**, pp. 25.

Kalwitzki, T., R. Müller, H. Rothgang, R. Runte and R. Unger (2015). 'BARMER GEK Pflegereport 2015'.

Katz, S., T. D. Downs, H. R. Cash and R. C. Grotz (1970). 'Progress in development of the index of ADL', *The gerontologist*, vol. **10**(**1 Part 1**), pp. 20-30.

LaPlante, M. P. (2010). 'The classic measure of disability in activities of daily living is biased by age but an expanded IADL/ADL measure is not', *The Journals of Gerontology Series B: Psychological Sciences and Social Sciences*, vol. **65**(**6**), pp. 720-732.

Lawton, M. P. and E. M. Brody (1969). 'Assessment of older people: self-maintaining and instrumental activities of daily living', *The gerontologist*, vol. **9**(**3**).

Molloy, G. J., H. M. McGee, D. O'Neill and R. M. Conroy (2010). 'Loneliness and Emergency and Planned Hospitalizations in a Community Sample of Older Adults', *Journal of the American Geriatrics Society*, vol. **58**(**8**), pp. 1538-1541.

Nazroo, J. (2017). 'Class and health inequality in later life: Patterns, mechanisms and implications for policy', *International Journal of Environmental Research and Public Health*, vol. **14**(**12**), pp. 1533.

Paaßen, G. (2012). 'Pflegereform 2012', in (Editor Ed.)^Eds.), *Book Pflegereform 2012*, City: Pflegestufe.info.

Peña-Longobardo, L. M., J. Oliva-Moreno, S. García-Armesto and C. Hernández-Quevedo (2016). 'The Spanish long-term care system in transition: Ten years since the 2006 Dependency Act', *Health policy*, vol. **120**(**10**), pp. 1177-1182.

Rapp, T., P. Chauvin and N. Sirven (2015). 'Are public subsidies effective to reduce emergency care? Evidence from the PLASA study', *Social Science & Medicine*, vol. **138**, pp. 31-37.

Silverstein, M., X. Chen and K. Heller (1996). 'Too much of a good thing? Intergenerational social support and the psychological well-being of older parents', *Journal of Marriage and the Family*, pp. 970-982.

1. On the reliability of self-reported health-conditions see, e.g., [Bound (1991)](#_ENREF_5), [Baker et al. (2004)](#_ENREF_2), [Dwyer and Mitchell (1999)](#_ENREF_12), [LaPlante (2010)](#_ENREF_17) and [Chan et al. (2012)](#_ENREF_9). [↑](#footnote-ref-2)
2. SHARE respondents are asked not to report difficulties that are expected to last less than three months. [↑](#footnote-ref-3)
3. SHARE respondents are asked not to report difficulties that are expected to last less than three months. [↑](#footnote-ref-4)
4. SHARE respondents are asked not to report difficulties that are expected to last less than three months. [↑](#footnote-ref-5)
5. A: The individual performs the task spontaneously, habitually, completely and correctly alone. B: The individual can perform the task alone, yet not spontaneously, and/or correctly and/or habitually and/or completely. C: The individual cannot perform, requires assistance or must have someone else’s help to do the activity. [↑](#footnote-ref-6)
6. SHARE respondents are asked not to report difficulties that are expected to last less than three months. [↑](#footnote-ref-7)
7. Details are available at [Dupourqué et al. (2012)](#_ENREF_11). A free AGGIR simulator is available at <http://www.ibou.fr/aggir/> [↑](#footnote-ref-8)
8. [Paaßen (2012)](#_ENREF_21) [↑](#footnote-ref-9)
9. The scale from 0 to 3 has the following meaning: 0 – Independent; 1- Mostly independent; 2 – Mostly dependent; 3 - Dependent [↑](#footnote-ref-10)
10. Law 36/2006 (*Ley de Dependencia*) - Act 39/2006, on the Promotion of Personal Autonomy and Care for Dependent persons [↑](#footnote-ref-11)
